# Systematic evaluation of error rates and causes in short samples in next-generation sequencing

**DOI:** 10.1038/s41598-018-29325-6

**Published:** 2018-07-19

**Authors:** Franziska Pfeiffer, Carsten Gröber, Michael Blank, Kristian Händler, Marc Beyer, Joachim L. Schultze, Günter Mayer

**Affiliations:** 1 University of Bonn, LIMES Institute, Chemical Biology, Gerhard-Domagk-Str. 1, 53121 Bonn, Germany; 2 AptaIT GmbH, Am Klopferspitz 19A, 82152 Planegg, Germany; 3 University of Bonn, LIMES Institute, Genomics and Immunoregulation, Carl-Troll-Str. 31, 53115 Bonn, Germany; 4 German Center for Neurodegenerative Diseases (DZNE) and University of Bonn, Platform for Single Cell Genomics and Epigenomics, Sigmund-Freud-Str. 25, 53127 Bonn, Germany; 5 DZNE, Molecular Immunology in Neurodegeneration, Sigmund-Freud-Str. 27, 53127 Bonn, Germany; 6 Center of Aptamer Research and Development, Gerhard-Domagk-Str. 1, 53121 Bonn, Germany

## Abstract

Next-generation sequencing (NGS) is the method of choice when large numbers of sequences have to be obtained. While the technique is widely applied, varying error rates have been observed. We analysed millions of reads obtained after sequencing of one single sequence on an Illumina sequencer. According to our analysis, the index-PCR for sample preparation has no effect on the observed error rate, even though PCR is traditionally seen as one of the major contributors to enhanced error rates in NGS. In addition, we observed very persistent pre-phasing effects although the base calling software corrects for these. Removal of shortened sequences abolished these effects and allowed analysis of the actual mutations. The average error rate determined was 0.24 ± 0.06% per base and the percentage of mutated sequences was found to be 6.4 ± 1.24%. Constant regions at the 5′- and 3′-end, e.g., primer binding sites used in *in vitro* selection procedures seem to have no effect on mutation rates and re-sequencing of samples obtains very reproducible results. As phasing effects and other sequencing problems vary between equipment and individual setups, we recommend evaluation of error rates and types to all NGS-users to improve the quality and analysis of NGS data.

## Introduction

**Figure 1:**
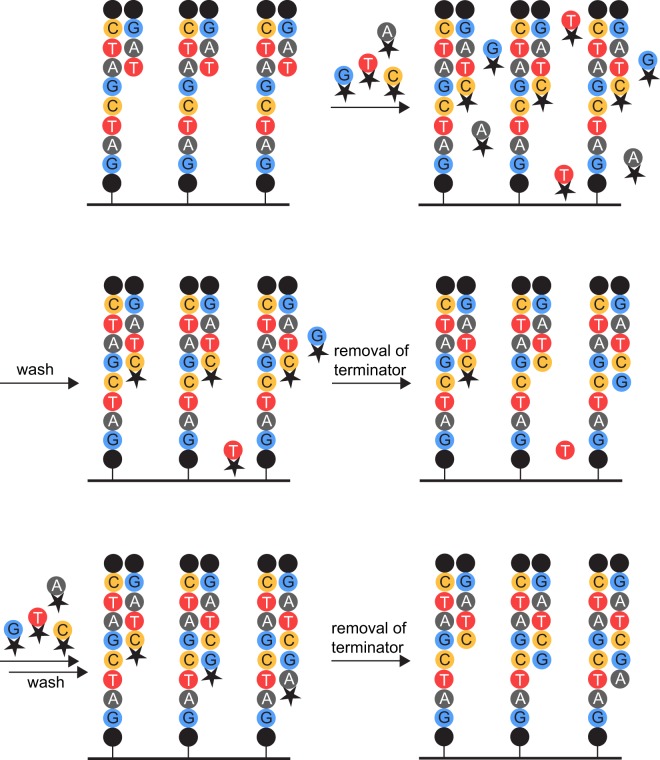
Origin of phasing effects. Depiction of the sequencing-by-synthesis approach. The black dots represent the sequencing primers. The terminator (black star) on the deoxynucleoside triphosphates (dNTPs) prevents the addition of the subsequent nucleotide to the growing DNA strand. The left strand depicts a post-phased sequence, the right strand a pre-phased one. The middle strand represents the state without phasing effects of any kind. If non-incorporated nucleotides remain after incorporation of the next nucleotide (upper right) and washes (middle left), removal of the terminator allows their addition to the growing strand (middle right, right strand). The resulting strand will subsequently be pre-phased. If the removal of the terminator is not complete (middle right, left strand), no nucleotide can be incorporated during the next sequencing cycle (lower left, left strand). The resulting strand will subsequently be post-phased.

The last decade has seen a steady increase in the use of next-generation sequencing (NGS) in all fields of biology due to the high sequence output and significantly reduced cost^1^. Alongside this development, it was discovered that the rates and types of errors depend on the sequencing method and platform used^2^. One of the most widely used sequencing techniques is sequencing-by-synthesis. The average error rate of this approach is reported to be 0.1% per nucleotide, most of which are single nucleotide substitutions^2^. In addition, the technique causes intrinsic errors: colour or laser cross-talk, cross-talk between adjacent clusters, phasing, and dimming^3–5^. Colour cross-talk results from the overlay of excitation and emission spectra between different fluorophores used for readout of the incorporated bases^4^. Once that has been corrected for, cross-talk between adjacent clusters due to the same reason still remains problematic^5^. Phasing describes two phenomena, both of which result in single sequences being out of phase with the rest of the cluster: Pre-phasing occurs if two (or more) nucleotides are incorporated in one cycle, because the flow-cell was not flushed adequately and non-incorporated nucleotides remained even after the terminator was removed and could therefore be incorporated. Post-phasing is caused by the incomplete removal of the terminator, leading to the sequence lagging behind the rest of the cluster (Fig. 1)^6^. Completely irremovable terminators as well as laser damage to the DNA strands lead to a decrease in the number of sequences sequenced in one cluster and therefore dimming of its fluorescent readout^4^. The base calling software Bustard encompasses an error correction for phasing events that assumes constant phasing rates^7^. Other methods improved on this by taking the surrounding nucleotides into account^7,8^ or adapting the algorithm on a run-by-run basis that can e.g., incorporate cycle-wise variations in cross-talk^4^. In addition to those technique-intrinsic errors, mutations result from PCR-errors during sample preparation and sequencing^2,9^. The investigation of overlaps (of paired end sequences^10–12^ or duplex-DNA^13^) can be used to decrease the error rate by rejecting bases that are not complementary on both strands. Mutations that occur during sequencing or due to one of the other problems as mentioned above can be analysed with indices or barcodes, whose error rates can be closely monitored^11,14–16^. In addition, quality assessment of single sequences has become pivotal enough that algorithms to determine sensible cut-off values for Phred scores for the data-set of interest are available^17^.

All these methods have in common that they were established for the determination of errors in sequences longer than the single NGS reads. Nonetheless, NGS is also used for the analysis of *in vitro* selections of aptamers, where the single read is long enough to cover the entire sequence of interest and no prior knowledge of the sequence is available^18–20^. While different analysis tools have been described^12,21–23^, no error analysis in the context with systematic evolution of ligands by exponential enrichment (SELEX) has been reported. We therefore aimed for a thorough error description and analysis of samples that are prepared analogous to *in vitro* selection samples: An index-PCR is used to add barcodes to the 5′- and 3′-end of the sequences to allow multiplexing of 12 samples in a single flow-cell. After adaptor-ligation, the samples are purified by agarose-gel extraction and quantified for NGS using qPCR^24^.

Our study showed that phasing effects were a major contributor to our initial error rates. Omission of shortened sequences allowed the exclusion of phased sequences and the determination of 0.25% per base as the real error rate. In addition, sequencing of identical samples seems to be well reproducible. We propose these findings to be important to increase the awareness of sequencing-specific problems like phasing effects and actual error rates during NGS and thereby support the well-informed use of NGS in the future.

## Results

### Effect of sample preparation

In order to investigate the effect of sample preparation on the error rate, we analysed the NGS results of the sequence of C12, a GFP-binding aptamer selected from a DNA library chemically modified by click-chemistry^25^. All templates were synthesized using the canonical set of nucleotides.

The index-PCR was performed with either PWO (Pyrococcus woesei) or Taq (Thermus aquaticus) polymerase. For C12_T_w/o, the template was synthesized including the indices. Therefore, no index-PCR was performed. After index-PCR, all samples were mixed, eliminating other steps as reason for differences between samples.

Analysed were both the percentage of mutated sequences as well as the average mutation per base, called error rate. No variations of the frequency of mutated sequences between the samples can be detected, not even for the sample prepared omitting the index-PCR. The error rate for C12_T_w/o, which has not been prepared by index PCR, is slightly lower than C12_T_Taq with C12_T_PWO showing the highest error rate (Table 1, Supplementary Figs S1–S3).

**Table 1:** Frequency of mutations in differentially prepared C12-samples.

sample name	EdU/T in template	DNA polymerase for index-PCR	mutated sequences [%]	non-mutated sequence [%]	error rate [%] (mean ± SD)	number of analysed sequences
C12_T_PWO	T	PWO	12.23	87.77	3.04 ± 1.87	1,119,179
C12_T_Taq	T	Taq	12.47	87.53	2.85 ± 1.75	3,416,163
C12_T_w/o	T	none^a^	12.43	87.57	2.55 ± 1.83	1,872,807
C12_EdU	EdU^b^	PWO	32.02	67.98	6.15 ± 4.01	4,593,685

^a^Oligo was solid-phase synthesized including the indices.

^b^Due to solid-phase synthesis of template, 20% of EdUs are oxidized to KdU^26,27^.

**Figure 2:**
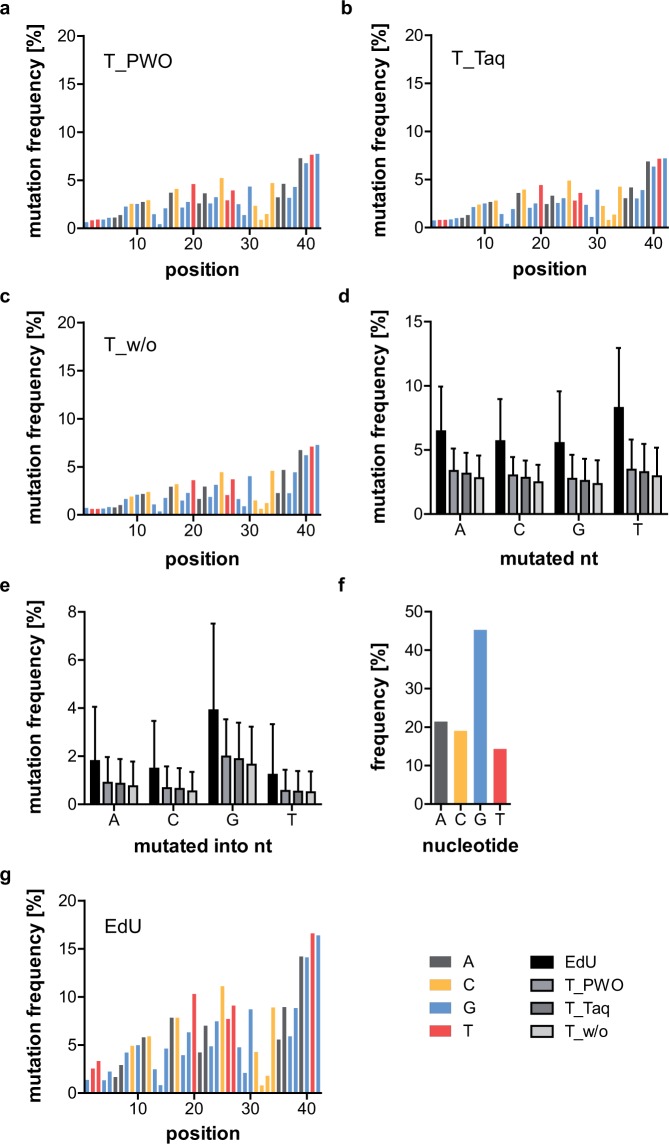
Mutation analysis of C12-samples. Mutation frequency of T_PWO- (**a**), T_Taq- (**b**), and T_w/o-samples (**c**) at each position of the random region. Denoted in colour is the original nucleotide at the respective position. Only minor variations between the different samples are visible. The mutation frequency increases from start to end of the random region in all samples. (**d**) Average mutation frequency of the four different nucleotides. EdU vs. T_PWO, T_Taq, and T_w/o p = 0.0286 (Mann-Whitney tests, two-tailed, preliminary Kruskal-Wallis test: p = 0.0132. n = 9, 8, 19, and 6 for A, C, G, and T, respectively). The remaining tests were non-significant. (**e**) Average mutation frequency with which mutations converted the original nucleotide into the denoted nucleotide. The Kruskal-Wallis test showed no significant differences between samples (n = 33, 34, 23, and 36 for A, C, G, and T, respectively). For both d and e, the EdU-sample shows the highest overall mutation frequency (significantly so for d), followed by T_PWO, T_Taq, and T_w/o with only a minor decrease in mutation frequency between the T-samples. Given is the mean and SD of each sample. (**f**) Frequency of the different nucleotides in the random region of the non-mutated C12-sequence. (**g**) Mutation frequency of C12_EdU at each position of the random region. Denoted in colour is the original nucleotide at the respective position. The mutation frequencies are much higher than those of the other C12-samples. As before, the mutation frequency increases from start to end of the random region.

Figure 2a–c show the mutation frequency for each position of all three samples. A clear increase over the length of the random region can be detected, resulting in an increase by a factor of about 10 from start to end. The average mutation frequency of the four original nucleotides is presented in Fig. 2d, while Fig. 2e shows the average mutation frequency with which the original nucleotide was converted into the denoted one. As for the error rates, samples prepared with Taq polymerase show a slightly lower mutation frequency for all nucleotides than those prepared with PWO polymerase. As expected, samples prepared without index-PCR show the lowest mutation frequency. Nonetheless, the differences are not significant. The average mutation frequency of the original nucleotide into the denoted one (Fig. 2e) of all four samples was found to reflect the nucleotide distribution of the original sequence (Fig. 2f). To investigate this correlation, we designed sequences with a repetitive random region.

### Effect of nucleobase-modifications

Before analysing the repetitive sequences, we wanted to investigate the effect of nucleobase-modifications on error rates in NGS. The template of C12_EdU was synthesized on the solid-phase with 5′-ethinyl-deoxyuridine (EdU) instead of thymidine. Due to the work-up procedures, about 20% of the EdU were converted to the ketone by-product (KdU) during deprotection, which might have an effect on PCR-fidelity^26,27^.

In comparison with the other C12-samples, all of which contained only the canonical nucleobases in the (PCR-)template, both the percentage of mutated sequences as well as the error rate are clearly increased for C12_EdU (32 and 6%, respectively, in contrast to about 12 and 3% for the samples containing thymidine) (Table 1, Supplementary Fig. S4). The same increase in mutation frequency can be detected when analysing the mutation rate from and into each of the four nucleotides separately, but the difference is only significant for the mutation rate from the different nucleotides (Fig. 2d,e). Although the absolute error values are higher than those of the non-EdU C12 sequences at every position, a similar increase in mutation rates over the length of the random region can be detected (Fig. 2g).

### Analysis of repetitive sequences

**Figure 3:**
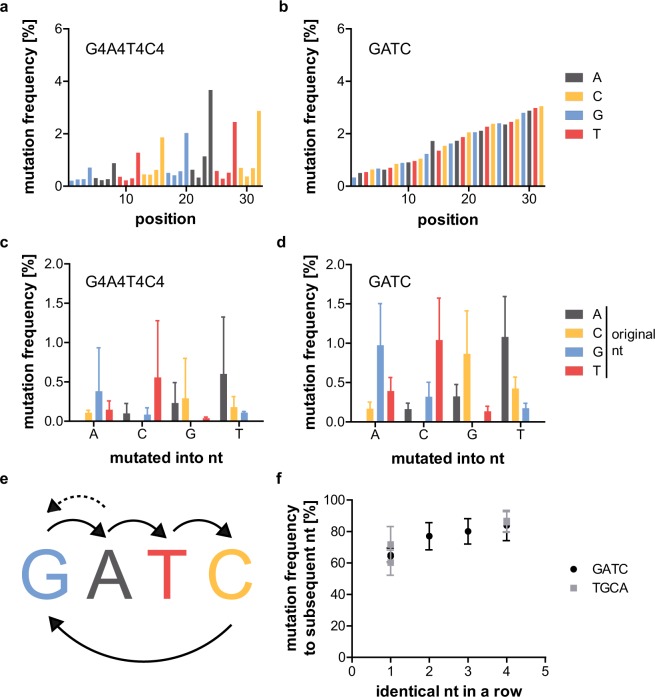
Mutation analysis of GATC-samples. Mutation frequency of G4A4T4C4- (**a**) and GATC-samples (**b**) at each position of the random region. Denoted in colour is the original nucleotide at the respective position. The GATC-sample shows a steady increase in mutation frequency from start to end of the random region. While the same trend is visible for the G4A4T4C4-sample, the mutation rate of the last of each of the four nucleotide blocks is much higher than the one of the first three nucleotides. Average mutation frequency (and standard deviation) with which mutations converted the original nucleotide into the denoted nucleotide for the G4A4T4C4- (**c**) and GATC-sample (**d**). Arrows in (**e**) indicate the most frequent conversions, with the dotted arrow valid only for the G4A4T4C4-sample. The most frequent mutations convert one nucleotide to the subsequent one. The indicated conversions occur with a significance of p ≤ 0.0174 for GATC (t-tests, two-tailed, preliminary one-way ANOVA: p = <0.0001, 0.0002, <0.0001, and 0.0007 for mutated into T, A, C, and G, respectively, n = 8). The conversions are non-significant for G4A4T4C4 (Kruskal-Wallis test, n = 8). (**f**) Frequency with which a nucleotide mutates to the subsequent nucleotide for all samples with 1 to 4 consecutive identical nucleotides. A clear increase in mutation frequency to the subsequent nucleotide can be seen with an increasing number of consecutive identical nucleotides. One consecutive identical nucleotide vs. four p = 0.0294 (Mann-Whitney test, two-tailed, n = 31 and 7 for 1 and 4 nucleotides in a row, respectively). Given is the mean and SD for each sample.

Table 2 describes the analysed repetitive sequences. GATC and G4A4T4C4 could not be sequenced by NGS as the sense and antisense strands could not be properly annealed (data not shown). Both initially analysed repetitive sequences use the primer binding sites of the FT2-library^25^. Their error rate and frequency of mutated sequences is lower than for the C12-samples (about 1.5 and 8%, respectively, compared to 3 and 12% for the C12-samples). FT2_G4A4T4C4 has a lower error rate, but a higher frequency of mutated sequences than FT2_GATC (Table 3 and Supplementary Figs S5 and S6). This can be explained when analysing the mutation frequency of each position of the random region: The first three nucleotides of each four-nucleotide block of FT2_G4A4T4C4 have a very low mutation frequency, while the last nucleotide of each block has a relatively high one. As before with the C12-samples, both samples show an increase in mutation frequency over the length of the random region by a factor of about 10 (Fig. 3a,b). The analysis of which nucleotides are mutated into which nucleotides (Fig. 3c,d) shows clear preferences for specific conversions. These have been outlined in Fig. 3e. Preferentially, the mutations seem to occur from one nucleotide to the subsequent one. This would also explain the low mutation frequency of the first three nucleotides of the four-nucleotide blocks of FT2_G4A4T4C4 (Fig. 3a) and the fact that the nucleotide composition of C12 is represented by the graph depicting the frequency with which a nucleotide mutates to the denoted one (Fig. 2e). The analysis of the percentage of a nucleotide mutating to the subsequent one is summarized in Table 4. As a completely random mutation would be represented by 33.3% of one nucleotide mutating into the subsequent one, the percentages ranging from 64 to 84% are significantly increased for all samples. While they do not vary much between the different C12-samples, the percentages for FT2_GATC (64%) are lower than for FT2_G4A4T4C4 (84%). To test if this finding correlates with the amount of identical consecutive nucleotides, FT2_G2A2T2C2 and FT2_G3A3T3C3 were also analysed (Supplementary Figs S7 and S8). In addition, the variants FT2-TGCA and FT2-T4G4C4A4 were sequenced to evaluate if the order of the nucleotides affects mutation rates (Supplementary Figs S9 and S10). These experiments revealed that the mutation frequency to the subsequent nucleotide increases steadily (from about 65 to 85%) with the number of identical consecutive nucleotides for all tested samples and is independent of the nucleotide order (Fig. 3f).

**Table 2:** Repetitive sequences.

sample name	index number^24^	index	primer sites from library	random region
GATC	10	TAGCTT	—	(GATC)_16_
G4A4T4C4	9	GATCAG	—	(GGGGAAAATTTTCCCC)_4_
FT2_GATC	11	GGCTAC	FT2^25^	(GATC)_8_
FT2_GATC_II	11	GGCTAC	FT2	(GATC)_8_
FT2_G4A4T4C4	12	CTTGTA	FT2	(GGGGAAAATTTTCCCC)_2_
FT2_G4A4T4C4_II	12	CTTGTA	FT2	(GGGGAAAATTTTCCCC)_2_
FT2_G2A2T2C2	6	GCCAAT	FT2	(GGGAAATTTCCC)_2_GGGAAATT
FT2_G3A3T3C3	5	ACAGTG	FT2	(GGAATTCC)_4_
FT2-TGCA	8	ACTTGA	FT2	(TGCA)_8_
D3-TGCA	10	TAGCTT	D3^33^	(TGCA)_8_
FT2-T4G4C4A4	7	CAGATC	FT2	(TTTTGGGGCCCCAAAA)_2_
D3-T4G4C4A4	9	GATCAG	D3	(TTTTGGGGCCCCAAAA)_2_

**Table 3:** Frequency of mutations in repetitive sequences.

sample name	mutated sequences [%]	non-mutated sequence [%]	error rate [%] (mean ± SD)	number of analysed sequences
FT2_GATC	8.44	91.56	1.63 ± 0.82	10,059,713
FT2_GATC_II	6.62	93.38	1.48 ± 0.78	2,332,475
FT2_G4A4T4C4	10.87	89.13	0.83 ± 0.83	8,235,942
FT2_G4A4T4C4_II	10.15	89.85	0.83 ± 0.83	7,288,615
FT2_G2A2T2C2	11.33	88.67	1.54 ± 0.96	2,301,791
FT2_G3A3T3C3	11.66	88.34	1.46 ± 1.08	6,265,796
FT2-TGCA	10.94	89.06	2.18 ± 1.16	7,441,266
D3-TGCA	7.27	92.73	1.09 ± 0.56	429,868
FT2-T4G4C4A4	10.79	89.21	0.92 ± 0.90	1,956,098
D3-T4G4C4A4	10.90	89.10	0.87 ± 0.97	5,930,886

**Table 4:** Frequency of mutation to subsequent nucleotide.

sample name	mutation to subsequent nt [%] (mean ± SD)
C12_EdU	76.0 ± 14.80
C12_T_PWO	73.2 ± 17.14
C12_T_Taq	74.1 ± 18.34
C12_T_w/o	76.5 ± 15.14
FT2_GATC	64.3 ± 3.85
FT2_GATC_II	65.0 ± 4.49
FT2-TGCA	60.2 ± 7.98
D3-TGCA	71.7 ± 11.45
FT2_G2A2T2C2	77.2 ± 8.65
FT2_G3A3T3C3	80.1 ± 8.07
FT2_G4A4T4C4	83.8 ± 9.63
FT2_G4A4T4C4_II	83.8 ± 9.63
FT2-T4G4C4A4	86.0 ± 6.57
D3-T4G4C4A4	86.6 ± 6.81

### Reproducibility of sequencing data and influence of the sequence of the primer binding sites on mutation rates

**Figure 4:**
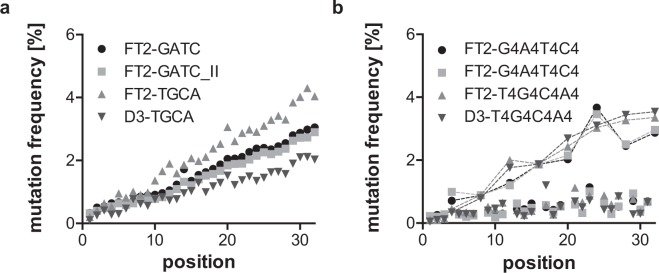
Mutation analysis of samples with repetitive sequences. Mutation frequency of samples with one (**a**) and four consecutive identical nucleotides (**b**) at each position of the random region. As before, the samples with one consecutive identical nucleotide show a steady increase in mutation frequency from start to end of the random region, while the samples with four consecutive identical nucleotides show this trend only for the last of each four nucleotide blocks. The repeated samples (FT2-GATC(_II) and FT2-G4A4T4C4(_II)) show very high similarity. While FT2-TGCA shows a slightly higher mutation rate than FT2-GATC and D3-TGCA shows a slightly lower one, the same trend cannot be seen for the samples with four consecutive identical nucleotides.

To evaluate the reproducibility of sequencing data, we reanalysed FT2-GATC and FT2- G4A4T4C4 (Supplementary Figs S11 and S12). The annealed dsDNA that had been prepared for the first sequencing was reused and adapter ligation, purification, and the sequencing repeated. Figure 4a,b as well as Tables 3 and 4 show that variations in error rate, mutation frequency, number of mutated sequences, and mutation frequency to the subsequent nucleotide are minimal even though the number of sequences obtained differ by a factor of 5 for FT2-GATC. We also evaluated the effect of changes of the primer binding sites on the mutation rates. For this, we tested two sequences with primer binding sites from both the FT2- and the D3-library (Table 2). While D3-TGCA shows slightly lower mutation frequencies and error rates, but a higher mutation frequency to the subsequent nucleotide than FT2-TGCA, no differences can be distinguished between D3-T4G4C4A4 and FT2-T4G4C4A4 (Fig. 4a,b, and Supplementary Figs S13 and S14 and Tables 3 and 4).

### Omission of shortened sequences excludes phasing effects

**Figure 5:**
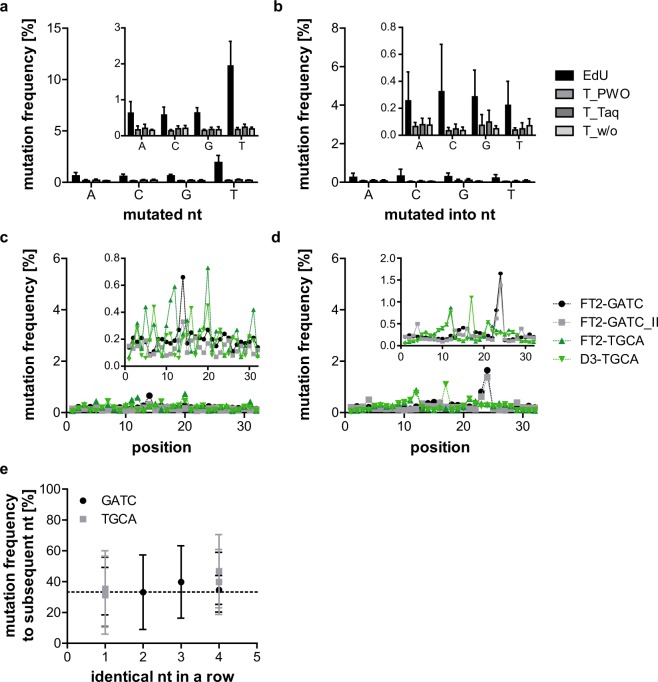
Mutation analysis after omission of shortened sequences. (**a**) Average mutation frequency of the four different nucleotides for the different C12-samples. EdU vs. T_Taq, and T_w/o p = 0.0286. T_PWO vs. T_Taq p = 0.0286 (Mann-Whitney tests, two-tailed, preliminary Kruskal-Wallis test: p = 0.0067, n = 9, 8, 19, and 6 for A, C, G, and T, respectively). The remaining tests were non-significant. (**b**) Average mutation frequency with which mutations converted the original nucleotide into the denoted nucleotide for the different C12-samples. EdU vs. T_PWO, T_Taq, and T_w/o p = 0.0286 (Mann-Whitney tests, two-tailed, preliminary Kruskal-Wallis test: p = 0.026, n = 33, 34, 23, and 36 for A, C, G, and T, respectively). The remaining tests were non-significant. For both a and b, the mutation frequencies are severely reduced upon omission of shortened sequences. While the EdU-sample still shows the significantly highest overall mutation frequencies, no clear trend is discernible for the samples containing T instead of EdU. Given is the mean and SD of each sample. Mutation frequency of samples with one (**c**) and four consecutive identical nucleotides (**d**) at each position of the random region. No increase in mutation frequency can be observed after omission of shortened sequences. Instead, the mutation frequency increases for certain samples at certain positions. The repeated samples (FT2-GATC(_II) and FT2-G4A4T4C4(_II)) show relatively high similarity, as is also the case for the FT2/D3-TGCA- and FT2/D3-T4G4C4A4-samples. While the larger graphs in (**a**–**d**) share the scale of similar graphs from Figs 1–3 to simplify comparisons before and after omission of shortened sequences, the smaller zoom-ins are scaled to allow a detailed view of the respective analysis. (**e**) Frequency with which a nucleotide mutates to the subsequent nucleotide for all samples with 1 to 4 consecutive identical nucleotides. No clear increase of mutation to the subsequent nucleotide can be discerned with increasing number of consecutive identical nucleotides. The average mutation frequency of 33.3% is indicated with a horizontal line and all values roughly correspond to this average. The difference between samples containing one and four identical nucleotides in a row is no longer significant (Mann-Whitney test, two-tailed, n = 31 and 7 for 1 and 4 nucleotides in a row, respectively). Given is the mean and SD for each sample.

As the increase in mutation frequencies over the length of the sequences and the high mutation rates to the subsequent nucleotide could be identified in all samples and are probably due to phasing effects, we aimed to exclude these from the analysis. Since the employed base calling software ‘Bustard’ should correct for phasing effects, additional software solutions like AYB^4^ did not seem promising. We therefore evaluated the 26 most abundant sequences in different samples and realised that the sequences containing pre-phasing effects are shortened (Supplementary Tables S1–S14). Obviously, the shortening of sequences may also result from deletions as these cannot be differentiated based on the sequencing data. The omission of the shortened sequences led to a strong decrease in percentage of mutated sequences and error rates for all investigated samples (Fig. 5a,b, and Supplementary Figs S15–S28 and Table 5). Also, the average mutation frequency of the original nucleotide into the denoted one of the C12-samples no longer reflects the nucleotide distribution of the original sequence (Figs 2f and 5a). While C12_EdU still shows the highest mutation frequency of all C12-samples, no clear trend in mutation rates can be seen for the three differentially prepared C12_T_samples (Fig. 5a,b). The omission of shortened sequences also led to a complete disappearance of the previously observed increase in mutation frequency over the length of the sequence for all samples (Fig. 5c,d). Instead of this clear trend, single mutations occur at seemingly random positions. Not only the repeated FT2-GATC(_II) and FT2-G4A4T4C4(_II)-samples, but also the samples with different primer binding sites (D3/FT2-TGCA and –T4G4C4A4) show similar hotspots for mutations. The mutation frequency to the subsequent nucleotide dropped to around the expected 33.3% and was now independent of the number of identical consecutive nucleotides (Fig. 5e, Tables 5 and 6). Table 6 summarizes the changes upon omission of the shortened sequences. The number of analysed sequences is reduced by an average of 5.2% and the non-mutated sequences increased by 5.6%. In contrast, the error rate dropped by 79%. All these were very clear indications that we had omitted the majority of mutated sequences created by pre-phasing without excluding a high percentage of sequences. We therefore re-analysed the samples to identify the ‘real’ error rates in NGS. C12_EdU still shows much increased mutation frequencies in comparison with all other samples (error rate 0.8%). As mentioned before, this is probably due to increased PCR-errors due to the EdU/KdU in the template. If C12_EdU is excluded, the average error rate of all other samples is 0.24 ± 0.06% per base and the average percentage of mutated sequences 6.4 ± 1.24%.

**Table 5:** Frequency of mutations if shortened sequences are omitted.

Sample name	Mutated sequences [%]	Non-mutated sequence [%]	Error rate [%] (mean ± SD)	Mutation to subsequent nt [%] (mean ± SD)	Number of analysed sequences
C12_EdU	19.31	80.69	0.81 ± 0.57	37.0 ± 16.87	3,869,868
C12_T_PWO	4.85	95.15	0.15 ± 0.06	44.7 ± 18.29	1,032,398
C12_T_Taq	5.72	94.28	0.19 ± 0.08	46.2 ± 21.52	3,171,344
C12_T_w/o	5.43	94.57	0.18 ± 0.07	35.4 ± 15.04	1,734,189
FT2_GATC	5.66	94.34	0.20 ± 0.09	33.8 ± 15.38	9,763,653
FT2_GATC_II	3.93	96.07	0.14 ± 0.05	33.4 ± 22.45	2,267,079
FT2_G4A4T4C4	7.96	92.04	0.28 ± 0.28	34.6 ± 9.37	7,975,576
FT2_G4A4T4C4_II	7.12	92.88	0.25 ± 0.23	39.6 ± 19.39	7,051,464
FT2_G2A2T2C2	7.73	92.27	0.29 ± 0.16	33.2 ± 24.18	2,211,912
FT2_G3A3T3C3	7.44	92.56	0.26 ± 0.14	39.8 ± 23.50	5,979,814
FT2-TGCA	7.23	92.77	0.24 ± 0.16	35.3 ± 24.73	7,143,566
D3-TGCA	5.41	94.59	0.18 ± 0.11	31.2 ± 25.28	421,388
FT2-T4G4C4A4	7.21	92.79	0.25 ± 0.17	46.8 ± 23.74	1,880,590
D3-T4G4C4A4	7.65	92.35	0.26 ± 0.22	39.8 ± 21.08	5,722,279

**Table 6:** Change upon omission of shortened sequences.

sample name	Δ number of analysed sequences [%]	Δ non-mutated sequences [%]	Δ error rate [%]	mutation to subsequent nt: deviation from 33.3% w/ w/o shortened sequences	
C12_EdU	−15.76	18.70	−86.83	42.7	3.7
C12_T_PWO	−7.75	8.41	−95.07	39.9	11.4
C12_T_Taq	−7.17	7.71	−93.33	40.8	12.9
C12_T_w/o	−7.40	7.99	−92.94	43.2	2.1
FT2_GATC	−2.94	3.04	−87.73	31.0	0.5
FT2_GATC_II	−2.80	2.88	−72.97	31.7	0.1
FT2_G4A4T4C4	−3.16	3.26	−66.27	26.9	1.3
FT2_G4A4T4C4_II	−3.25	3.37	−69.88	38.4	6.3
FT2_G2A2T2C2	−3.90	4.06	−81.17	43.9	−0.1
FT2_G3A3T3C3	−4.56	4.78	−43.48	46.8	6.5
FT2-TGCA	−4.00	4.17	−88.99	50.5	2.0
D3-TGCA	−1.97	2.01	−83.49	50.5	−2.1
FT2-T4G4C4A4	−3.86	4.01	−72.83	52.7	13.5
D3-T4G4C4A4	−3.52	3.65	−70.11	53.3	6.5
average	−5.15	5.57	−78.93	42.31	4.61

C12_EdU was excluded for calculation of the averages.

**Figure 6:**
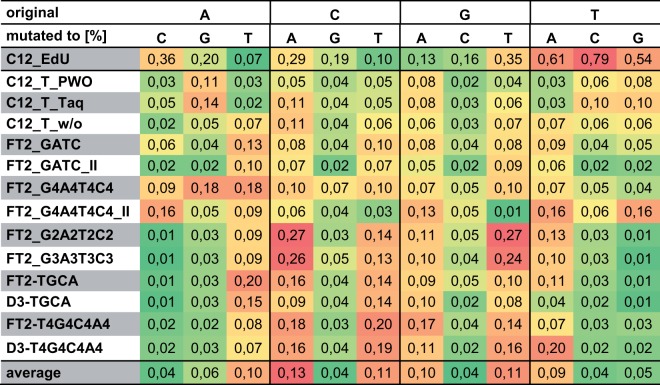
Conversion between nucleotides after omission of shortened sequences. The percentage of conversion from one specific nucleotide to another in each analysed sample is indicated by colour. High conversion rates are marked in red, low rates in green. The colouring was performed separately for (A) C12_EdU, (B) the other samples, and (C) the average. C12_EdU was excluded for calculation of the averages. Clear preferences for certain conversions are visible: While A to C, C to G, T to C, T to C, and T to G are particularly rare, C to A followed by C to T, G to T, A to T, and G to A are the most abundant conversions over all samples. More general, C and G have higher mutation rates than A and T. In contrast, C and G are most rarely mutated into, with high mutation rates to T and A. FT2-G2A2T2C2 and FT2-G3A3T3C3 have the highest mutation rates of all samples with conversions from C to A and G to T.

The percentage of mutations substituting one for another nucleotide after omission of the shortened sequences is depicted in Fig. 6. Again, C12_EdU shows the highest mutation rates of all samples and was therefore excluded for the calculation of the averages. In addition, the colouring according to the values was done separately for C12_EdU. For C12_EdU, the highest mutation rates are from thymidine (T) to – in order from high to low – cytidine (C), adenine (A), and guanine (G) with 0.79, 0.61, and 0.54%, respectively. Of the other samples, FT2-G2A2T2C2 and FT2-G3A3T3C3 showed the highest mutation rates from C to A (with 0.27 and 0.26%, respectively) and G to T (with 0.27 and 0.24%, respectively). Overall (after exclusion of C12_EdU), C followed by G have the highest mutation rates (average 0.093 and 0.083%, respectively) with T and A coming last (average 0.06 and 0.067, respectively). When it comes to the nucleotides that are most often mutated into, the exact opposite can be observed: T and A show the highest values (average 0.107%), while C and G are extremely low (average 0.04 and 0.05, respectively). In average, the most frequent substitution is C to A (0.13%), followed by C to T, and G to T (both 0.11%). The least frequent mutations occur from A to C, C to G, G to C, and T to C (all 0.04%).

### Effect of omission of shortened sequences on SELEX samples

**Figure 7:**
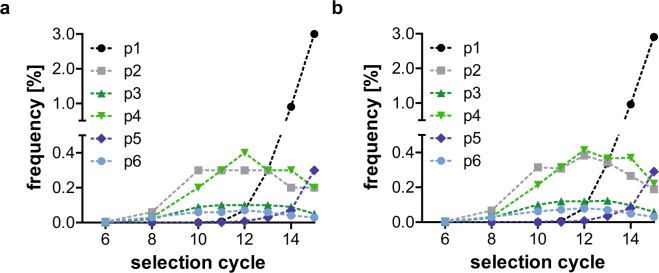
Frequency of SELEX patterns before and after omission of shortened sequences. Frequency of four SELEX patterns before (**a**) and after (**b**) omission of shortened sequences in different selection cycles. Selection cycles lower than cycle 6 were excluded as the values were too low to be visible. Only slight differences are apparent, while the overall trends stay unchanged.

In order to ascertain the effect of the exclusion of shortened sequences on samples from *in vitro* selection procedures, we reanalysed samples from a selection for nucleobase-modified GFP-aptamers^25^. Figure 7 shows the frequency of four different patterns (sequence families that were clustered using relative information entropy) over different selection cycles. Only slight differences before (Fig. 7a) and after (Fig. 7b) omission of shortened sequences can be observed. The general trends as well as absolute frequencies do not change.

## Discussion

While a multitude of studies use NGS, the number of publications concerning error rates, descriptions, and corrections are still rather low. We aimed to give an insight into error rates and types in the widely used sequencing-by-synthesis approach.

### Phasing

Sequencing of single sequences led us to the conclusion that different outcomes we were seeing (increase in error rate over the length of the sequence, high mutation rates of nucleotides to the subsequent ones (Figs 2–4)) were based on pre-phasing effects. The increase in error rate over the length of the sequence was also reported in previous papers that used sequencing-by-synthesis sequencers, even though the extent of the phenomenon was not as pronounced as in our samples^6,28–30^. While optimisation of the washing cycles during sequencing might be able to reduce phasing, the relevant parameters can only be changed in the program’s code which goes along with warranty loss.

Omission of shortened sequences leads to a reduction in error rates of in average 79%, while the number of analysed sequences and the percentage of non-mutated sequences only changed by roughly 5% each (Table 6). This is a clear indication that the sequences we removed from the analysis were the major contributor to the error rate, as the remaining 95% of sequences only reflected 20% of the error rate. Since pre-phasing means that the insertion of one nucleotide is not visible, all subsequent nucleotides that differ from the previous will be analysed as mutated. Therefore, a low amount of sequences suffering from pre-phasing effects has a huge impact on the error rate. Only reduction or complete ablation of these sequences allows insights into the real mutation rates.

Even though published software shows phasing correction that is improved in contrast to the Bustard algorithm, the percentage of perfect reads does not exceed 77%, which is far lower than the in average 94% we gained by removing all shortened sequences from the analysis^4^. Obviously, not all samples allow the omission of shortened sequences. If the sequence length is unknown, new algorithms are needed. Nonetheless, samples whose length is known, in particular in samples from *in vitro* selection procedures, and who are found to suffer from phasing-effects, will benefit from this solution.

### Effects of sample preparation

Quite often, errors in NGS are attributed to PCR-errors during sample preparation or the sequencing process^2,9,14^. While we could not evaluate the second, we investigated the index-PCR during sample preparation by using three differentially prepared samples: prepared with Taq, or PWO DNA-polymerase, and without any index-PCR. According to the manufacturer, PWO should have a 10x higher fidelity than Taq polymerase. After correcting our error analysis for phasing-effects by omission of shortened sequences, we could not detect any significant differences between the three differentially prepared samples (Fig. 5a,b). In contrast, Oyola *et al*., who sequenced AT-rich sequences, found the PCR-free preparation to be significantly better than any of the polymerases tested, which included AccuPrime Taq HiFi (a hot-start Taq polymerase mixed with e.g., a proofreading enzyme, which should have a 9x higher fidelity than Taq alone), but not Taq alone or PWO^31^.

In addition to the three above-mentioned samples, we also tested a sample with the same sequence, but containing EdU instead of thymidine. Even though it was prepared for NGS like C12_T_PWO, the error rates are significantly higher (Fig. 5a,b). We assume that this results from higher error rates during PCR-amplification because of the artificial base. Taken together, we conclude that influences of PCR-preparation for NGS are negligible if the samples themselves are not problematic for PCR, as found for AT-rich sequences^31^ and C12_EdU (this study). We would like to point out that we cannot exclude errors during solid-phase synthesis that are on par with PCR-errors during index-PCR. As the different fidelity of the two polymerases should have led to differences between the two samples that have been prepared by PCR, we nonetheless deduce that such errors must be marginal.

### Reproducibility of sequencing data and the impact of primer binding sites on mutation rates

Before (Fig. 4, Tables 3 and 4) as well as after omission of shortened sequences (Fig. 5c,d, Tables 5 and 6), no significant differences between the samples that were sequenced twice (FT2-GATC(_II) and FT2-G4A4T4C4(_II)) were observed. After exclusion of shortened sequences, the samples show similar hotspots for point mutations. While this might be attributed to errors of the template that occurred during solid-phase synthesis, the same is true for FT2-TGCA and D3-TGCA as well as FT2-T4G4C4A4 and D3-T4G4C4A4 (Fig. 5c,d). As those do not share the same template, the likeliest explanation for these error hotspots is that they are sequence-dependent. This would also concur with the fact that the samples with one consecutive identical nucleotide show more hotspots (Fig. 5c) than those with four (Fig. 5d). As no differences between FT2-TGCA/-T4G4C4A4 and D3-TGCA/-T4G4C4A4 can be distinguished, the primer binding sites seem to have no discernible effect on error rates. In addition, our experiments reveal that NGS data from SELEX-like libraries and sequences seem to be well reproducible.

### Error analysis after exclusion of phasing-effects

After omission of shortened sequences and exclusion of C12_EdU due to its high mutation rates, the mean error rate was found to be 0.24 ± 0.06%. Table 7 gives an overview of published error rates that have been obtained with different Illumina sequencers. The error rate observed by us is in the lower range compared with the published ones. This might be due to the exclusion of shortened sequences and therefore of sequences generated by phasing effects. Even before omission of shortened sequences, the average error rate (without C12_EdU) of 1.56 ± 0.81% fits within the published values, although it is on the higher end. Concerning the most and least abundant substitutions, the high mutation rates for T in C12_EdU support the hypothesis that EdU, which is replaced by T during the index-PCR, is responsible for the increased error rates of this sample. All other conversion values are only slightly higher or on par with those of the other samples.

**Table 7:** Published error rates on Illumina sequencers.

Publication	Instrument	Error rate [%]	Comments
Fox *et al*.^2^	HiSeq2000	0.1	
Fox *et al*.^2^	MiSeq	0.1	
Dohm *et al*.^29^	1 G	0.3	at the start of sequence, increases due to phasing effects
May *et al*.^34^	MiSeq	0.21–2.6	depending on the reference sequence; substitutions only
Kelley *et al*.^30^	not disclosed	0.5–2	

Due to the similarity of the emission spectra of the used fluorophores, AC and GT are most frequently miscalled for each other in Illumina sequencing^6^. This is partly represented by our data, where C to A and G to T are among the most abundant substitutions. Nonetheless, A to C and T to G are among the rarest mutations, even though A to C should be the most frequent one according to other publications^29,30^. The least frequent mutation according to Dohm *et al*. occurs from C to G, which is also one of the least frequent ones in our dataset^29^. As we see deviations of up to a factor of 10 even between re-sequenced, but otherwise identical samples (FT2-GATC(_II) and FT2-G4A4T4C4(_II)), larger datasets seem to be needed to enable satisfying explanations.

### Omission of shortened sequences in SELEX samples

The frequencies of four different patterns in several selection cycles only change marginally upon omission of shortened sequences (Fig. 7). This is probably due to the fact that we analyse patterns instead of single sequences. Sequences resulting from phasing events will still end up in the same pattern as the original sequence. As phased and therefore shortened sequences are part of every pattern, the absolute frequency values do not change either.

### Recommendation of the authors

Our mutation data showed a huge impact of phasing effects that we could exclude by omission of all shortened sequences. While the problem is known in the literature^6,28–30^, it does not seem to be as prominent and problematic for every sequencing setup. We therefore recommend everybody using NGS routinely to sequence e.g., some of the repetitive sequences published here to gain insight into the error types and rates of your own setup.

Regarding the use of NGS for analysis of SELEX procedures, we would like to point out that the omission of shortened sequences might also result in the omission of binding sequences as shortened sequences can also be native to the enriched library. If the shortening is strong enough to be apparent on agarose gels of the enriched library, our solution can obviously not be used. We recommend computational solutions to exclude phasing effects if that is the case. In addition, analyses of single sequences will suffer much more strongly from both actual mutations during sequencing as well as sequencing errors than analyses based on sequence families like the patterns presented here or those consisting of sequences that only differ by a low number (1 to 5) of mutations.

## Material and Methods

All oligodeoxynucelotides were obtained from Ella Biotech GmbH, Martinsried, Germany.

### Sample preparation

Samples were prepared and sequenced in several different runs: 1) C12_EdU, 2) C12_T_wo, C12_T_PWO, and C12_T_Taq, 3) GATC, G4A4T4C4, FT2_GATC, and FT2_G4A4T4C4, 4) FT2_GATC_II, FT2_G4A4T4C4_II, FT2_G2A2T2C2, FT2_G3A3T3C3, FT2-TGCA, D3-TGCA, FT2-T4G4C4A4, and D3-T4G4C4A4, 5) GFP-SELEX samples.

All samples were prepared according to Tolle *et al*. with the exception of the index-PCR^24^. The index-PCR was only performed for C12_EdU, C12_T_PWO, and the GFP-SELEX samples (using PWO-DNA polymerase (Genaxxon, Ulm, Germany)), as well as C12_T_Taq (using Taq DNA polymerase (in house production) and large Klenow fragment (NEB, Ipswich, USA) according to the manufacturer’s instructions for blunt end generation). All other sequences were commercially obtained as both sense and anti-sense strand including the indices and annealed as follows: 100 pmol of both strands were mixed in 40 mM Tris, pH 7.9. After heating at 95 °C for 5 min, the strands were slowly cooled down to 4 °C in 30 min (0.05 °C/s). Successful annealing was determined by agarose gel electrophoresis.

The thereby gained dsDNA was purified from an agarose gel with a Gel and PCR cleanup kit (Macherey-Nagel, Düren, Germany) and ligated with an adaptor that allows hybridization to the sequencing flow cell according to the manufacturer’s instructions (TruSeq DNA PCR-Free (LT) sample preparation kit, Illumina, San Diego, USA). After agarose gel purification, the libraries were quantified using the KAPA library quantification kit for Illumina libraries according to manufacturer’s instructions on a Roche LightCycler 480.

### Next-generation sequencing

Libraries were clustered at 7 pM supplemented with 20% 10 pM PhiX on a SR HiSeq Rapid Cluster Kit v2 flow cell or at 1.1 pM supplemented with 20% 1.8 pM PhiX using a NextSeq 500/550 High Output v2 kit (75 cycles) and sequenced over 76 base pairs and 7 index bases on a HiSeq1500 or NextSeq500 system, respectively (Illumina, San Diego, USA). Sequencing data were demultiplexed using bcl2fastq2 v2.18.0.12.

### NGS-analysis

Analysis of NGS-data was accomplished with the software tool COMPAS^22,32^. Sequences were directly parsed from FASTQ files. For this purpose, sample specific bar codes where used to assign sequences to the respective datasets. In the next step, the random region of each sequence was defined by teaching the COMPAS software the flanking, constant primer regions. The relative distribution of the A, C, G, T nucleotide building blocks over the random region was calculated for all datasets.

For the GFP-SELEX samples, patterns were identified in silico in datasets of selection cycles 1, 2, 4, 6, 8, 10, 11, 12, 13, 14, and 15. For each cycle, in the first step, similar sequences were clustered by using relative information entropy as a measure to group sequences to patterns of related sequences. In the second step, sequences of each cluster were counted to calculate the relative frequency of the entire cluster as well as for each monoclonal sequence of each cluster. To trace the enrichment behavior of defined patterns, COMPAS was used to calculate the relative frequency of patterns p1, p2, p3, p4, p5, and p6 in all datasets of selection cycles.

For the omission of shortened sequences, only sequences of the correct length or longer were considered for the respective analyses.

### Mutational analysis

The frequency of mutated sequences was calculated by setting the overall number of sequences for that sample to 100%. The percentage was calculated for the number of non-mutated sequences obtained and subtracted from 100% to gain the ‘frequency of mutated sequences’.

The mutation rate per nucleotide was calculated from the nucleotide distribution by subtracting the frequency of the correct nucleotide at a specific position from 1. The average and standard deviation of the mutation frequency per nucleotide of a specific nucleotide was given as ‘mutated nt’. The overall average and standard deviation of all mutated nucleotides is the ‘error rate’.

To calculate the average and standard deviation of the ‘mutated into nt’, all frequencies of that nucleotide at all positions where it was not the original nucleotide were taken into consideration. The frequencies at the specific positions were also used to determine the frequency of mutation from one nucleotide into another specific nucleotide and to calculate the average and standard deviation of the mutation to the subsequent nucleotide (for each position: 100/(1 − frequency correct nucleotide) * frequency subsequent nt).

### Statistical analysis

Normality of the datasets was tested using Shapiro-Wilk normality test. The normally distributed datasets were analysed by one-way ANOVA to establish the existence of significant differences between all datasets followed by two-tailed t-tests to evaluate the significances between two specific datasets. The not normally distributed datasets were analysed by Kruskal-Wallis test for initial determination of significant differences between all datasets followed by two-tailed Mann-Whitney tests to evaluate the significances between two specific datasets. For all tests, alpha was set to 0.05.

### Data availability

The datasets generated and analysed during this study are available from the corresponding author on request.

## Electronic supplementary material

Supplementary Information
