# Supplementary material for: Systematic evaluation of error rates and causes in short samples in next-generation sequencing
Source: Sci Rep. 2018 Jul 19;8:10950. doi: 10.1038/s41598-018-29325-6 (PMC6053417; doi:10.1038/s41598-018-29325-6)
Supplement: Supplementary file 1 — Supplementary Information [file 41598_2018_29325_MOESM1_ESM.pdf]

Supplementary Data

Systematic evaluation of error rates and causes in short samples in next-generation sequencing

Franziska Pfeiffer, Carsten Gröber, Michael Blank, Kristian Händler, Marc Beyer, Joachim L. Schultze, and Günter Mayer*

For all tables, yellow indicates substitutions, green insertions, and turquoise deletions.

Table S1: Original sequence and 25 most frequent mutations of C12_T_PWO

Sequence	Sequence count
**GTTGGAAGCGACGGGACGGTAAGGCTTGGGCCCCAAGGAGTG**	**982317**
GGTTGGAAGCGACGGGACGGTAAGGCTTGGGCCCCAAGGAGTG	4753
TTGGAAGCGACGGGACGGTAAGGCTTGGGCCCCAAGGAGTG	4240
GTTGGAAGCGACGGGACGGTAGGGCTTGGGCCCCAAGGAGTG	3792
GTTGGAAGCGACGGGACGGTAAGGCTTGGCCCCAAGGAGTG	3722
GTTGGAAGCGACGGACGGTAAGGCTTGGGCCCCAAGGAGTG	3515
GTTGGAAGCGACGGGACGGTAAGGCTTGGGCCCAAGGAGTG	2732
GTGGAAGCGACGGGACGGTAAGGCTTGGGCCCCAAGGAGTG	2226
GTTGGAAGCGACGGGACGTAAGGCTTGGGCCCCAAGGAGTG	1970
GTTGGAAAGCGACGGGACGGTAAGGCTTGGGCCCCAAGGAGTG	1943
GTTGGAAGCGACGGGACGGTAGGCTTGGGCCCCAAGGAGTG	1886
GTTGAAGCGACGGGACGGTAAGGCTTGGGCCCCAAGGAGTG	1727
GTTGGAGCGACGGGACGGTAAGGCTTGGGCCCCAAGGAGTG	1711
GTTGGAAGCGACGGGGACGGTAAGGCTTGGGCCCCAAGGAGTG	1688
GTTGGGAAGCGACGGGACGGTAAGGCTTGGGCCCCAAGGAGTG	1641
GTTGGAAGCGACGGGACGGTAAGGCTGGGCCCCAAGGAGTG	1590
GTTGGAAGCGACGGGACGGTAAGGCTTGGGCCCCCAAGGAGTG	1412
GTTGGAAGCGACGGCGGTAAGGCTTGGGCCCCAAGGAGTG	1319
GTTGGAAGCGACGGGACGGTAAGGCTTGGGCCCCAAAGGAGTG	1301
GTTGGAAGCGACGGGACGGTAAGGCTTGGGCCCCAAGAGTG	1291
GTTGGAAGCGACGGGACGGTAAGCTTGGGCCCCAAGGAGTG	1239
GTTGGAAGCGACGGGACGGTAAGGCTGGGGCCCCAAGGAGTG	1193
GTTGGAAGCGACGGGACGGTAAGGCTTGGGCCCCAAGAAGTG	1137
GTTGGAAGCGACGGGACGGTGAGGCTTGGGCCCCAAGGAGTG	1123
GTTGGAAGCGACGGGAGGTAAGGCTTGGGCCCCAAGGAGTG	1085
GTTGGAAGCGACGGGACGGTAAGGTTGGGCCCCAAGGAGTG	1059

Table S2: Original sequence and 25 most frequent mutations of C12_T_Taq

Sequence	Sequence count
**GTTGGAAGCGACGGGACGGTAAGGCTTGGGCCCCAAGGAGTG**	**2990041**
GGTTGGAAGCGACGGGACGGTAAGGCTTGGGCCCCAAGGAGTG	13977
GTTGGAAGCGACGGGACGGTAGGGCTTGGGCCCCAAGGAGTG	12302
TTGGAAGCGACGGGACGGTAAGGCTTGGGCCCCAAGGAGTG	11493
GTTGGAAGCGACGGGACGGTAAGGCTTGGCCCCAAGGAGTG	11343
GTTGGAAGCGACGGACGGTAAGGCTTGGGCCCCAAGGAGTG	10431

GTTGGAAGCGACGGGACGGTAAGGCTTGGGCCCAAGGAGTG	7847
GTGGAAGCGACGGGACGGTAAGGCTTGGGCCCCAAGGAGTG	6353
GTTGGAAAGCGACGGGACGGTAAGGCTTGGGCCCCAAGGAGTG	6098
GTTGGAAGCGACGGGACGTAAGGCTTGGGCCCCAAGGAGTG	5541
GTTGGAAGCGACGGGGACGGTAAGGCTTGGGCCCCAAGGAGTG	5406
GTTGAAGCGACGGGACGGTAAGGCTTGGGCCCCAAGGAGTG	5179
GTTGGAGCGACGGGACGGTAAGGCTTGGGCCCCAAGGAGTG	5091
GTTGGAAGCGACGGGACGGTAGGCTTGGGCCCCAAGGAGTG	5059
GTTGGGAAGCGACGGGACGGTAAGGCTTGGGCCCCAAGGAGTG	4909
GTTGGAAGCGACGGGACGGTAAGGCTTGGGCCCCCAAGGAGTG	4721
GTTGGAAGCGACGGGACGGTAAGGCTGGGCCCCAAGGAGTG	4541
GTTGGAAGCGACGGGACGGTGAGGCTTGGGCCCCAAGGAGTG	4053
GCTGGAAGCGACGGGACGGTAAGGCTTGGGCCCCAAGGAGTG	3972
GTTGGAAGCGACGGGACGGTAAGGCTTGGGCCCCAAAGGAGTG	3948
GTTGGAAGCGACGGGACGGTAAGGCTGGGGCCCCAAGGAGTG	3914
GTTGGAGGCGACGGGACGGTAAGGCTTGGGCCCCAAGGAGTG	3772
GTTGGAAGCGACGGGACGGTAAGGCTTGGGCCCCAAGAGTG	3694
GTTGGAAGCGACGGGCGGTAAGGCTTGGGCCCCAAGGAGTG	3635
GTTGGAAGCGACGGGACGGTAAGCTTGGGCCCCAAGGAGTG	3560
GTTGGAAGCGACGGGACGGTAAGGCTTGGGCCCCAAGAAGTG	3536

Table S3: Original sequence and 25 most frequent mutations of C12_T_w/o

Sequence	Sequence count
**GTTGGAAGCGACGGGACGGTAAGGCTTGGGCCCCAAGGAGTG**	**1640067**
GTTGGAAGCGACGGGACGGTAAGGCTTGGGCCCAAGGAGTG	7565
GTTGGAAGCGACGGGACGGTAAGGCTTGGCCCCAAGGAGTG	5878
GGTTGGAAGCGACGGGACGGTAAGGCTTGGGCCCCAAGGAGTG	5444
TTGGAAGCGACGGGACGGTAAGGCTTGGGCCCCAAGGAGTG	4726
GTTGGAAGCGACGGACGGTAAGGCTTGGGCCCCAAGGAGTG	4683
GTTGGAAGCGACGGGACGGTAGGCTTGGGCCCCAAGGAGTG	4033
GTTGGAAGCGACGGGACGGTAAGCTTGGGCCCCAAGGAGTG	3930
GTTGGAAGCGACGGGACGGTAAGGCTTGGGCCCCAAGGAGTGC	3777
GTTGGAAGCGACGGGACGGTAAGGCTGGGCCCCAAGGAGTG	3700
GTTGAAGCGACGGGACGGTAAGGCTTGGGCCCCAAGGAGTG	3346
GTTGGAAGCGACGGGACGGAAGGCTTGGGCCCCAAGGAGTG	3342
GTTGGAAGCGACGGGACGGTAAGGCTTGGGCCCCAGGAGTG	3119
GTTGGAAGCGACGGGACGGTAAGGCTTGGGCCCCAAGGAGT	2986
GTTGGAAGCGACGGGACGGTAAGGCTTGGGCCCCAAGGATG	2715
GTTGGAAGCGACGGGACGGTAAGGCTTGGGCCACAAGGAGTG	2713
GTTGGAAGCGACGGGACGTAAGGCTTGGGCCCCAAGGAGTG	2659
GTTGGAAGCGACGGGACGGTAAGGTTGGGCCCCAAGGAGTG	2635
GTTGGAAGCGACGGGACGGTAAGGCTTGGGCCCCAAGAGTG	2609
GTTGGAGCGACGGGACGGTAAGGCTTGGGCCCCAAGGAGTG	2500
TTTGGAAGCGACGGGACGGTAAGGCTTGGGCCCCAAGGAGTG	2437
GTTGGAAGCGACGGGCGGTAAGGCTTGGGCCCCAAGGAGTG	1991
GTTGGAAAGCGACGGGACGGTAAGGCTTGGGCCCCAAGGAGTG	1989
GTGGAAGCGACGGGACGGTAAGGCTTGGGCCCCAAGGAGTG	1842
GTTGGGAAGCGACGGGACGGTAAGGCTTGGGCCCCAAGGAGTG	1839

GCTGGAAGCGACGGGACGGTAAGGCTTGGGCCCCAAGGAGTG	1740

Table S4: Original sequence and 25 most frequent mutations of C12_EdU

Sequence	Sequence count
**GTTGGAAGCGACGGGACGGTAAGGCTTGGGCCCCAAGGAGTG**	**3122777**
GTTGGAAGCGACGGGACGGTAAGGCCTGGGCCCCAAGGAGTG	51393
GTTGGAAGCGACGGGACGGTAAGGCTTGGGCCCCAAGGATG	42097
GTTGGAAGCGACGGGACGTAAGGCTTGGGCCCCAAGGAGTG	35342
TTGGAAGCGACGGGACGGTAAGGCTTGGGCCCCAAGGAGTG	29569
GTCGGAAGCGACGGGACGGTAAGGCTTGGGCCCCAAGGAGTG	27323
GCTGGAAGCGACGGGACGGTAAGGCTTGGGCCCCAAGGAGTG	25350
GTTGGAAGCGACGGGACGGTAAGGCATGGGCCCCAAGGAGTG	24557
GTAGGAAGCGACGGGACGGTAAGGCTTGGGCCCCAAGGAGTG	24291
GTTGGAAGCGACGGACGGTAAGGCTTGGGCCCCAAGGAGTG	23940
GGTTGGAAGCGACGGGACGGTAAGGCTTGGGCCCCAAGGAGTG	21405
GTTGGAAGCGACGGGACGGTAAGGCTTGGGCCCCAAGGAGG	20464
GTTGGAAGCGACGGGACGGTAAGGCTTGGGCCCAAGGAGTG	18605
GATGGAAGCGACGGGACGGTAAGGCTTGGGCCCCAAGGAGTG	18243
GTTGGAAGCGCCGGGACGGTAAGGCTTGGGCCCCAAGGAGTG	18183
GTTGGAAGCGACGGGCCGGTAAGGCTTGGGCCCCAAGGAGTG	17191
GTTGGAAGCGACGGGACGGTAAGGCTTGGGCCCCAAGAGTG	16764
GTTGGAAGCGACGGGACGGTAAGGCGTGGGCCCCAAGGAGTG	15361
GTTGGAAGCGACGGGACGGTAAGGCTAGGGCCCCAAGGAGTG	15273
GTTGGAAGCGACGGGACGGTAGGCTTGGGCCCCAAGGAGTG	15044
GTTGGAAGCGACGGGACGGTAAGGCTTGGGCCCCAAGGAGAG	14747
GTTGGAAGCGACGGGGACGGTAAGGCTTGGGCCCCAAGGAGTG	14639
GTGGAAGCGACGGGACGGTAAGGCTTGGGCCCCAAGGAGTG	14417
GTTGGAAGCGACGGGACGGTAAGGCTTGGCCCCAAGGAGTG	13760
GTTGAAGCGACGGGACGGTAAGGCTTGGGCCCCAAGGAGTG	13186
GTTGGAAGCGACGGGACGGTAAGGCTTTGGCCCCAAGGAGTG	13117

Table S5: Original sequence and 25 most frequent mutations of FT2_GATC

Sequence	Sequence count
**GATCGATCGATCGATCGATCGATCGATCGATC**	**9210580**
GATCGATCGATCGTTCGATCGATCGATCGATC	40756
GATCGATCGATCGATCGATCGATCGATC	16625
ATCGATCGATCGATCGATCGATCGATCGATC	15189
GATCGATCGATCGATCGATCGATCGATCGAT	12245
GATCGATCGATCGATTGATCGATCGATCGATC	11192
GATCGATCGATCGATCGATCGATAGATCGATC	10571
GATCGATCGATCGATCGTTCGATCGATCGATC	10186
GATCGATTGATCGATCGATCGATCGATCGATC	10022
GATCGATCGATCGCTCGATCGATCGATCGATC	9607
GATCGATCGATCTATCGATCGATCGATCGATC	9462
GATCGATCGATCGATCGATCGATCAATCGATC	9387
GATCGATCGATCGATCGTCGATCGATCGATC	8657
GATCGATCGATCGATCGATCGATCGATCGTTC	8628

GATTGATCGATCGATCGATCGATCGATCGATC	8511
GATCGATCGATCGATCGACGATCGATCGATC	8406
GATCGATCGATCGATCGATCGAACGATCGATC	8334
GATCGATCGATCGATCGATTGATCGATCGATC	8294
GATCGATCGATCGATCGATCGACGATCGATC	8159
GTTCGATCGATCGATCGATCGATCGATCGATC	8081
GATCGATCGATCGATCTATCGATCGATCGATC	7884
GATCGATCGATCGATCAATCGATCGATCGATC	7740
GATCGATCTATCGATCGATCGATCGATCGATC	7715
GATCGATCGATCGATCGATAGATCGATCGATC	7604
GATCGATCGATCGATCGAACGATCGATCGATC	7408
GATCGATCGATTGATCGATCGATCGATCGATC	7398

Table S6: Original sequence and 25 most frequent mutations of FT2_GATC_II

Sequence	Sequence count
**GATCGATCGATCGATCGATCGATCGATCGATC**	**2178012**
GATCGATCGATCGTTCGATCGATCGATCGATC	6052
GATCGATCGATCGATCGATCGATCGATCGATC	4924
ATCGATCGATCGATCGATCGATCGATCGATC	3450
GATCGATCGATCGATCGATCGATCGATCGAT	2870
GATCGATCGATCGATCTATCGATCGATCGATC	2324
GATCGATCGATCGATCGTTCGATCGATCGATC	2019
GATCGATCGATCGATCGTCGATCGATCGATC	2003
GATCTATCGATCGATCGATCGATCGATCGATC	1992
GATCGATCGATCGATTGATCGATCGATCGATC	1972
GATCGATCGATCGATCGACGATCGATCGATC	1948
GATCGATCGATCTATCGATCGATCGATCGATC	1942
GATCGATTGATCGATCGATCGATCGATCGATC	1921
GATCGATCGATCGATCGATCGATCGATCGTTC	1905
GATCGATCGATCGATCGATCGATAGATCGATC	1851
GATCGATCGATCGATCGATCGATCAATCGATC	1740
GATCGATCGATCGATCGATCGACGATCGATC	1721
GTTCGATCGATCGATCGATCGATCGATCGATC	1715
TATCGATCGATCGATCGATCGATCGATCGATC	1676
GATCGATCGATCGATCGATTGATCGATCGATC	1640
GATCGATCGATCGATCGATCGTCGATCGATC	1596
GATCGATCGATCGATGATCGATCGATCGATC	1594
GATCGATCGATCATCGATCGATCGATCGATC	1588
GATCGATCGATCGTCGATCGATCGATCGATC	1583
GATCGATCGATCGACGATCGATCGATCGATC	1577
GATTGATCGATCGATCGATCGATCGATCGATC	1565

Table S7: Original sequence and 25 most frequent mutations of FT2_G4A4T4C4

Sequence	Sequence count
**GGGGAAAATTTTCCCCGGGGAAAATTTTCCCC**	**7340975**
GGGGAAAATTTTCCCCGGGGAAAGTTTTCCCC	62115
GGGGAAAATTTTCCCCGGGGAAATTTTTCCCC	29603

GGGGAAAATTTTCCCCGGGGAAACTTTTCCCC	28885
GGGAAAATTTTCCCCGGGGAAAATTTTCCCC	27528
GGGGAAAATTTTCCCCGGGGAAAATTTTCCC	26734
GGGGAAAATTTTCCCGGGGAAAATTTTCCCC	25567
GGGGAAAATTTTCCCCGGGGAATATTTTCCCC	25363
GGGGAAAATTTTCCCCGGGAAAATTTTCCCC	22129
GGGGAAAATTTTCCCCGGGGAAGATTTTCCCC	20528
GGGGAAAATTTCCCCGGGGAAAATTTTCCCC	18488
GGGGAAAATTTTCCCCGGGGAAATTTTCCCC	14789
GGGGAAATTTTCCCCGGGGAAAATTTTCCCC	13789
GGGGAAAATTTTCCCCGGGGAAAATTTCCCC	13618
GGGGAAAATTTTCCCGGGGGAAAATTTTCCCC	12188
GGGGAAAATTTTCCACGGGGAAAATTTTCCCC	11202
GGGGAAAATTTTCCTCGGGGAAAATTTTCCCC	10651
GGGGAAAATTTTCCCCGGGGAACATTTTCCCC	9828
GGGGAAAATTTTCCCCGGGGTAAATTTTCCCC	9817
GGGGAAAATTTTCACCGGGGAAAATTTTCCCC	9097
GGGGAAAATTTTCTCCGGGGAAAATTTTCCCC	8683
GGGGAAAATTTTCCCCGAGGAAAATTTTCCCC	8659
GGGGATAATTTTCCCCGGGGAAAATTTTCCCC	8512
TGGGAAAATTTTCCCCGGGGAAAATTTTCCCC	8250
GGGGAAAATTTTCCCCGTGGAAAATTTTCCCC	8185
GGGGAAAATTTTCCCCGGGGATAATTTTCCCC	8177

Table S8: Original sequence and 25 most frequent mutations of FT2_G4A4T4C4_II

Sequence	Sequence count
**GGGGAAAATTTTCCCCGGGGAAAATTTTCCCC**	**6549166**
GGGGAAAATTTTCCCCGGGGAAAGTTTTCCCC	45606
GGGGAAAATTTTCCCCGGGGAAAATTTTCCC	25874
GGGAAAATTTTCCCCGGGGAAAATTTTCCCC	25002
GGGGAAAATTTTCCCCGGGGAAATTTTTCCCC	24447
GGGGAAAATTTTCCCGGGGAAAATTTTCCCC	23700
GGGGAAAATTTTCCCCGGGAAAATTTTCCCC	20951
GGGGAAAATTTTCCCCGGGGAAACTTTTCCCC	20084
GGGTAAAATTTTCCCCGGGGAAAATTTTCCCC	19289
GGGGAAAATTTTCCCCGGGGAATATTTTCCCC	18918
GGGGAAAATTTCCCCGGGGAAAATTTTCCCC	16348
GGGGAAAATTTTCCCCGGGGAAAATTTTACCC	15108
GGGGAAAATTTTCCCCGGGGAAGATTTTCCCC	14713
GGGGAAAATTTTCCCCGGGGAAATTTTCCCC	13451
GGGGAAAATTTTCCCCGGGGAAAATTTCCCC	13426
GGGGAAATTTTCCCCGGGGAAAATTTTCCCC	12803
GGGGAAAATTTTCCCGGGGGAAAATTTTCCCC	11949
GGGGAAAATTTTACCCGGGGAAAATTTTCCCC	11730
TGGGAAAATTTTCCCCGGGGAAAATTTTCCCC	8438
GGTGAAAATTTTCCCCGGGGAAAATTTTCCCC	8037
GGGGAAAATTTTCCCCGGGTAAAATTTTCCCC	7069
GGGGATAATTTTCCCCGGGGAAAATTTTCCCC	6921

GGGGAAAATTTTCACCGGGGAAAATTTTCCCC	6831
GGGGAAAATTTTCCCCGGGGAAAATTTTCACC	6576
GGGGAATATTTTCCCCGGGGAAAATTTTCCCC	6558
GGGGAAAATTTTCCCCGGGGAAAATTTTCCAC	6555

Table S9: Original sequence and 25 most frequent mutations of FT2_G2A2T2C2

Sequence	Sequence count
**GGAATTCCGGAATTCCGGAATTCCGGAATTCC**	**2040896**
GAATTCCGGAATTCCGGAATTCCGGAATTCC	6073
GGAATTCCGGAATTCCGTAATTCCGGAATTCC	5990
GGAATTCCGGAATTACGGAATTCCGGAATTCC	5539
GGAATTCCGGAATTCCGGAATTCCGGAATTC	5123
GGAATTCCGGAATTCCGGAATTCCGTAATTCC	5085
GGAATTTCGGAATTCCGGAATTCCGGAATTCC	5080
GGAATTCCGGAATTCCGGAATTCCGGAATTAC	4697
GGAATTCCGTAATTCCGGAATTCCGGAATTCC	4674
GGAATTCCGGAATTCCGGAATTACGGAATTCC	4548
GGAATTACGGAATTCCGGAATTCCGGAATTCC	4547
GTAATTCCGGAATTCCGGAATTCCGGAATTCC	4165
GGAATTCCGGAATTCGGAATTCCGGAATTCC	4057
GGAATTCCGGAATTCCGAATTCCGGAATTCC	3949
GGAATTCCGGAATTCAGGAATTCCGGAATTCC	3664
GGAATTCCGGAATTCCGGAATTCAGGAATTCC	3571
GGAATTCCGGAATTCCGGAATTCCGGAATTCA	3541
TGAATTCCGGAATTCCGGAATTCCGGAATTCC	3522
GGAATTCCGGAATTCCTGAATTCCGGAATTCC	3473
GGAATTCCTGAATTCCGGAATTCCGGAATTCC	3450
GGAATTCCGAATTCCGGAATTCCGGAATTCC	3374
GGAATTCCGGAATCCGGAATTCCGGAATTCC	3215
GGAATTCTGGAATTCCGGAATTCCGGAATTCC	3196
GGAATTCCGGAATTCCGGAATCCGGAATTCC	3180
GGAATTCCGGAATTCTGGAATTCCGGAATTCC	3161
GGAATTCCGGAATTCCGGATTCCGGAATTCC	3152

Table S10: Original sequence and 25 most frequent mutations of FT2_G3A3T3C3

Sequence	Sequence count
**GGGAAATTTCCCGGGAAATTTCCCGGGAAATT**	**5535182**
GGAAATTTCCCGGGAAATTTCCCGGGAAATT	21093
GGGAAATTTCCCGGGAAATTCCCGGGAAATT	19864
GGGAAATTTCGGGAAATTTCCCGGGAAATT	19079
GGGAAATTTCCCGGGATTTCCCGGGAAATT	18549
GGGAAATTTCCCGGGAAATTTACCGGGAAATT	17688
GGGAAATTTCCCGAAATTTCCCGGGAAATT	16754
GGGAAATTTCCAGGGAAATTTCCCGGGAAATT	16257
GGGAAATTTCCCGGGAAATTTCCAGGGAAATT	16206
GGGAAATTTCCCTGGAAATTTCCCGGGAAATT	15353

GGGAAATTTCCCGGGAAATTTCCGGGAAATT	14511
GGTAAATTTCCCGGGAAATTTCCCGGGAAATT	13353
GGGAAATTTCCCGGGAAATTTCCCGGAAATT	12584
TGGAAATTTCCCGGGAAATTTCCCGGGAAATT	12115
GGGAAATTTCCCGGTAAATTTCCCGGGAAATT	12041
GGGAAATTTCCCGGGAAATTTCCCTGGAAATT	11707
GGGAAATTCCCGGGAAATTTCCCGGGAAATT	11475
GGGAAATTTCCCGTGAAATTTCCCGGGAAATT	11190
GGGAAATTTCCCGGGAAATTTCCCGGGAATT	10863
GGGAAATTTCACGGGAAATTTCCCGGGAAATT	10839
GGGAAATTTCCCGGGAAATTTCACGGGAAATT	10796
GGGAAATTTCCCGGGAAATTTCCCGTGAAATT	10637
GGGAATTTCCCGGGAAATTTCCCGGGAAATT	10332
GTGAAATTTCCCGGGAAATTTCCCGGGAAATT	10015
GGGAAATTTACCGGGAAATTTCCCGGGAAATT	9290
GGGAAATTTCCCGGGAAATTTCCCGGTAAATT	8848

Table S11: Original sequence and 25 most frequent mutations of FT2_TGCA

Sequence	Sequence count
**TGCATGCATGCATGCATGCATGCATGCATGCA**	**6627344**
TGCATGCATGCATGCATGCTTGCATGCATGCA	45693
TGCATGCATGCTTGCATGCATGCATGCATGCA	36139
TGCAAGCATGCATGCATGCATGCATGCATGCA	26613
TGCATGCATGCATGCATGCATGCATGCA	24728
TGCATGCATGCATGCATGCATGCATGCATGAA	19114
TGCATGCATGAATGCATGCATGCATGCATGCA	18958
TGTATGCATGCATGCATGCATGCATGCATGCA	12451
TGCATGCATTCATGCATGCATGCATGCATGCA	11260
TGCATGTATGCATGCATGCATGCATGCATGCA	10812
TGCATGCATGCATGTATGCATGCATGCATGCA	10365
TGCATGCATGTATGCATGCATGCATGCATGCA	10306
TGCATGCATGCATGAATGCATGCATGCATGCA	9163
TGCATGCATGCATGCATGTATGCATGCATGCA	8532
TGCATGCATGCATGCATGCATGCATGTATGCA	7920
TGCATGCATGCATGCATGCATGTATGCATGCA	7487
TGCATGCATGCTGCATGCATGCATGCATGCA	7444
TGCATGCATGCATGCATGCATGCATGCATACA	7208
TGCATGCATCATGCATGCATGCATGCATGCA	7143
TGCATGCATGCATGCATGAATGCATGCATGCA	7044
TGCATGCATGCATGCATGCATGCATGAATGCA	6912
TGCATGAATGCATGCATGCATGCATGCATGCA	6769
TGAATGCATGCATGCATGCATGCATGCATGCA	6711
TGCATGCATGCATGCATGCATGATGCATGCA	6706
TGCATGCATGCATCATGCATGCATGCATGCA	6549
TGCATGCATGCATGCATGCATGCATACATGCA	6531

Table S12: Original sequence and 25 most frequent mutations of D3_TGCA

Sequence	Sequence count
**TGCATGCATGCATGCATGCATGCATGCATGCA**	**398611**
TGCATGCATGCATGCATGCTTGCATGCATGCA	1654
TGCATGCATGCATGCTTGCATGCATGCATGCA	1560
TGCATGTATGCATGCATGCATGCATGCATGCA	1190
TGCATGCATGCATGCATGCATGCATGCATGCA	1026
TGCATGCATGCATGCATGCATGTATGCATGCA	588
TGCATGCTTGCATGCATGCATGCATGCATGCA	555
TGCATGCATGCATGCATGTATGCATGCATGCA	545
TGCATGCATGCATGCATGCATGCATGCATACA	530
TGTATGCATGCATGCATGCATGCATGCATGCA	518
TGCATGCATGCATGCATGCATGCATACATGCA	498
TGCATGCATGTATGCATGCATGCATGCATGCA	459
TGCATGCATTCATGCATGCATGCATGCATGCA	455
TGAATGCATGCATGCATGCATGCATGCATGCA	418
TGCATGCATGCATGCATACATGCATGCATGCA	395
TGCATGCATGCATACATGCATGCATGCATGCA	393
TGCATGCATGCATGTATGCATGCATGCATGCA	392
TGCATGCATGCATGCATGCATACATGCATGCA	390
TGCATGAATGCATGCATGCATGCATGCATGCA	379
TGCATGCTGCATGCATGCATGCATGCATGCA	368
TGCATGCATGCATGAATGCATGCATGCATGCA	364
TGCATGCATGCATGCATTCATGCATGCATGCA	345
TGCATGCATGCATGCATGCATGCATGCATGAA	344
TGCATACATGCATGCATGCATGCATGCATGCA	335
TGCATGCATGCATGCATGAATGCATGCATGCA	332
TGCATGCATGCATGCATGCATGCATGAATGCA	306

Table S13: Original sequence and 25 most frequent mutations of FT2_T4G4C4A4

Sequence	Sequence count
**TTTTGGGGCCCCAAAATTTTGGGGCCCCAAAA**	**1745068**
TTTTGGGGCCCAAAAATTTTGGGGCCCCAAAA	9556
TTTTGGGGCCCCAAAATTTGGGGCCCCAAAA	7973
TTTTGGGGCCCCAAATTTTGGGGCCCCAAAA	7625
TTTTGGGGCCCCAAAATTTTGGGCCCCAAAA	7388
TTTTGGGGCCCAAAATTTTGGGGCCCCAAAA	6884
TTTTGGGCCCCAAAATTTTGGGGCCCCAAAA	4855
TTTTGGGGCCCCAAAATTTTGGGGCCCAAAA	4841
TTTTGGGGCTCCAAAATTTTGGGGCCCCAAAA	4576
TTTTGGGGCCTCAAAATTTTGGGGCCCCAAAA	4488
TTTTGGGGCCCCAAAATTTTGGGGCCCCAAA	4334
TTTGGGGCCCCAAAATTTTGGGGCCCCAAAA	4234
TTTTGGGGCCCCAAAATTTTTGGGCCCCAAAA	4229
TTTTGGGGCCCTAAAATTTTGGGGCCCCAAAA	4204
TTTTGGGGCCCCAAAATTTTGGGGCTCCAAAA	3987
TTTTGGGTCCCAAAATTTTGGGGCCCCAAAA	3946
TTTTGGGGCCCCAAAATTTTAGGGCCCCAAAA	3878
TTTTGGGGCCCCAAAATTTTGGAGCCCCAAAA	3860

TTTTGGGGCCCCAAAATTTTGAGGCCCCAAAA	3606
TTTTGGGGCCCCAAAATTTTGGGACCCCAAAA	3308
TTTTGGGGCCCCAAAATTTTGTGGCCCCAAAA	2968
TTTTGGGGCCACAAAATTTTGGGGCCCCAAAA	2940
TTTTGTGGCCCCAAAATTTTGGGGCCCCAAAA	2649
TTTTGGGGCCCCAAATTTTTGGGGCCCCAAAA	2524
TTTTGGGGCCCCAAAAATTTTGGGGCCCCAAAA	2469
TTTTGGGGCCCCAAAATTTTGGGGCCACAAAA	2430

Table S14: Original sequence and 25 most frequent mutations of D3_T4G4C4A4

Sequence	Sequence count
**TTTTGGGGCCCCAAAATTTTGGGGCCCCAAAA**	**5284258**
TTTTGGGGCCCCAAAAATTTGGGGCCCCAAAA	56226
TTTTGGGGCCCCAAATTTTGGGGCCCCAAAA	33501
TTTTGGGGCCCCAAAATTTGGGGCCCCAAAA	31991
TTTTGGGGCCCAAAAATTTTGGGGCCCCAAAA	19746
TTTTGGGGCCCCAAAATTTTGGGCCCCAAAA	18308
TTTTGGGGCCCCAAAATTTTGGGGCCCAAAA	18108
TTTTGGGGCCCAAAATTTTGGGGCCCCAAAA	16172
TTTTGGGGCCCCAAAATTTTTGGGCCCCAAAA	16010
TTTTGGGCCCCAAAATTTTGGGGCCCCAAAA	15538
TTTTGGGGCCCTAAAATTTTGGGGCCCCAAAA	14219
TTTTGGGGCTCCAAAATTTTGGGGCCCCAAAA	14016
TTTTGGGGCCTCAAAATTTTGGGGCCCCAAAA	13073
TTTTGGGGCCCCAAAATTTTGGGGCCCCAAA	12625
TTTTGGGGTCCCAAAATTTTGGGGCCCCAAAA	10980
TTTGGGGCCCCAAAATTTTGGGGCCCCAAAA	10697
TTTTGGGGCCACAAAATTTTGGGGCCCCAAAA	9072
TTTTGGGGCCCCAAAATTTTGTGGCCCCAAAA	8441
TTTTGTGGCCCCAAAATTTTGGGGCCCCAAAA	8378
TTTTGGGGCCCCAAAATTTTAGGGCCCCAAAA	7925
TTTTGGGGCCCCAAAATTTTGGGGCCACAAAA	7891
TTTTTGGGCCCCAAAATTTTGGGGCCCCAAAA	7708
TTTTGGGGCCCCAAAATTTTGGGACCCCAAAA	7669
TTTTGGTGCCCCAAAATTTTGGGGCCCCAAAA	7171
TTTTGGGGCCCCAAAATTTAGGGGCCCCAAAA	7018
TTTTGGGGCCCCAAAATTTTGAGGCCCCAAAA	6677

Figure S1

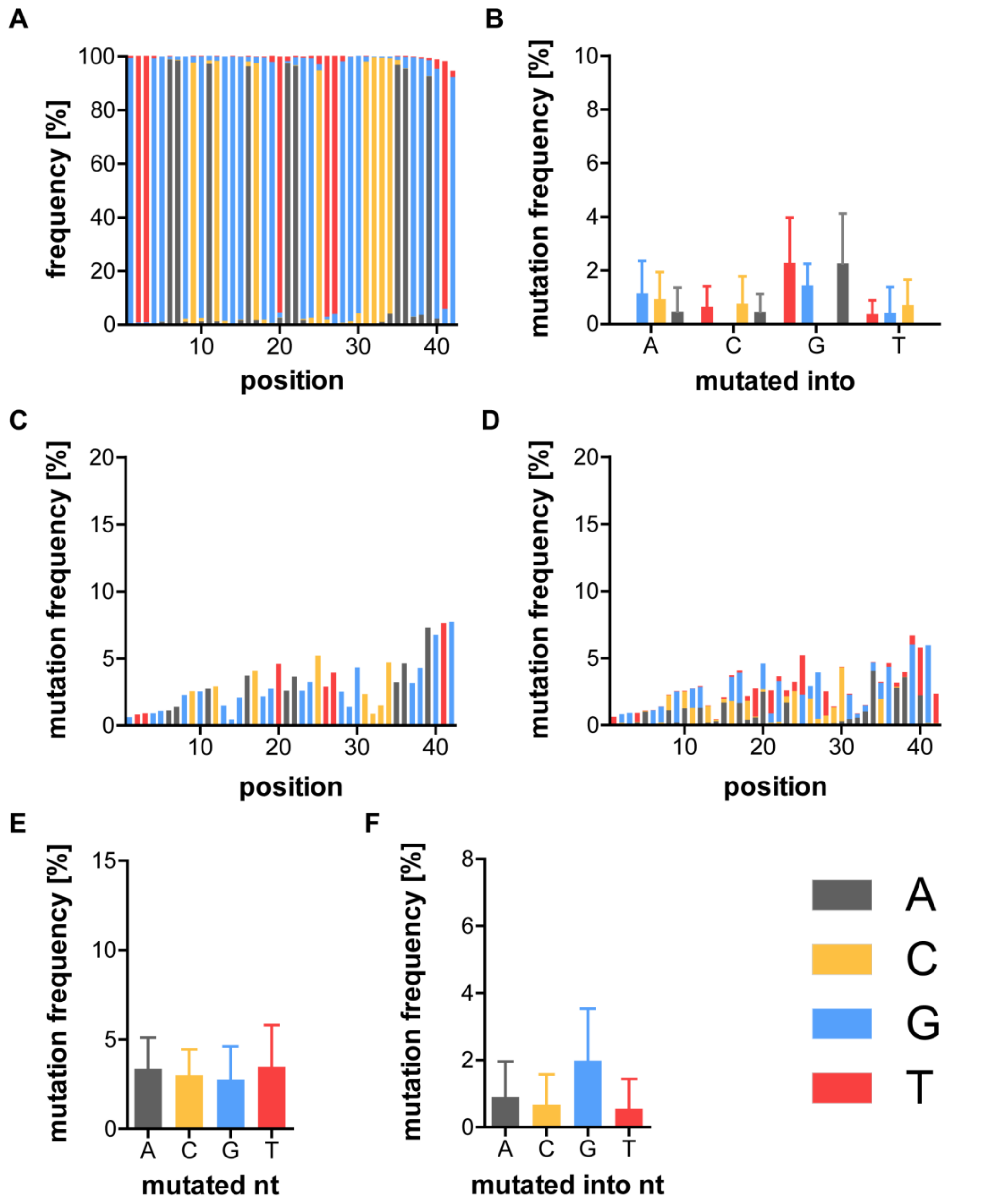

Figure S1: Mutation analysis of C12_T_PWO

(**A**) Frequency of the different nucleotides at all positions of the random region. (**B**) Frequencies with which one nucleotide (denoted by colour) mutates into another nucleotide (denoted on the x-axis). Depicted is the mean and SD. Mutation frequency at all positions of the random region of (**C**) the original nucleotide and (**D**) the nucleotide that has been mutated into. Mutation frequencies of the original nucleotides (**E**) and the nucleotides that has been mutated into (**F**). Depicted is the mean and SD.

Figure S2

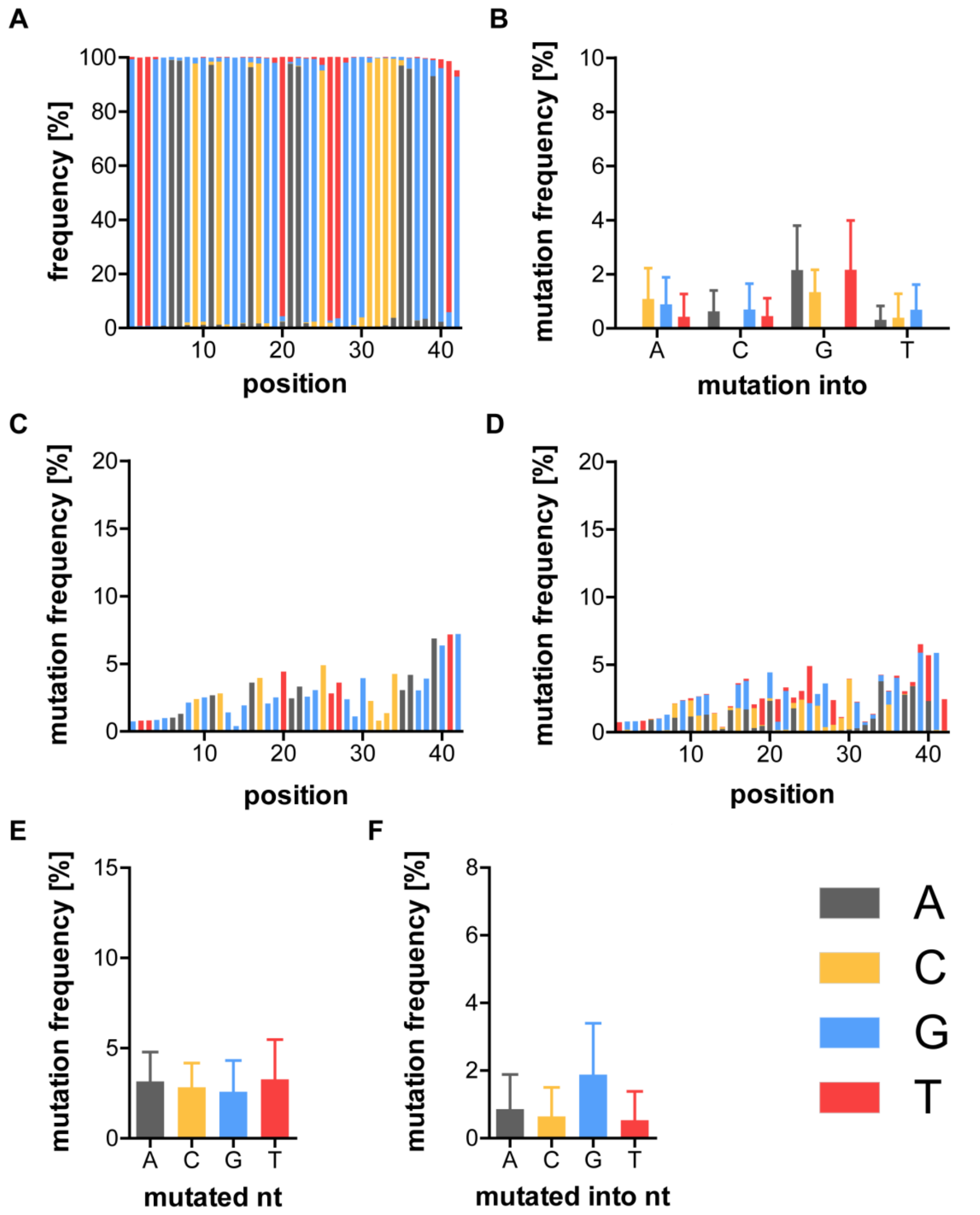

Figure S2: Mutation analysis of C12_T_Taq

(**A**) Frequency of the different nucleotides at all positions of the random region. (**B**) Frequencies with which one nucleotide (denoted by colour) mutates into another nucleotide (denoted on the x-axis). Depicted is the mean and SD. Mutation frequency at all positions of the random region of (**C**) the original nucleotide and (**D**) the nucleotide that has been mutated into. Mutation frequencies of the original nucleotides (**E**) and the nucleotides that has been mutated into (**F**). Depicted is the mean and SD.

Figure S3

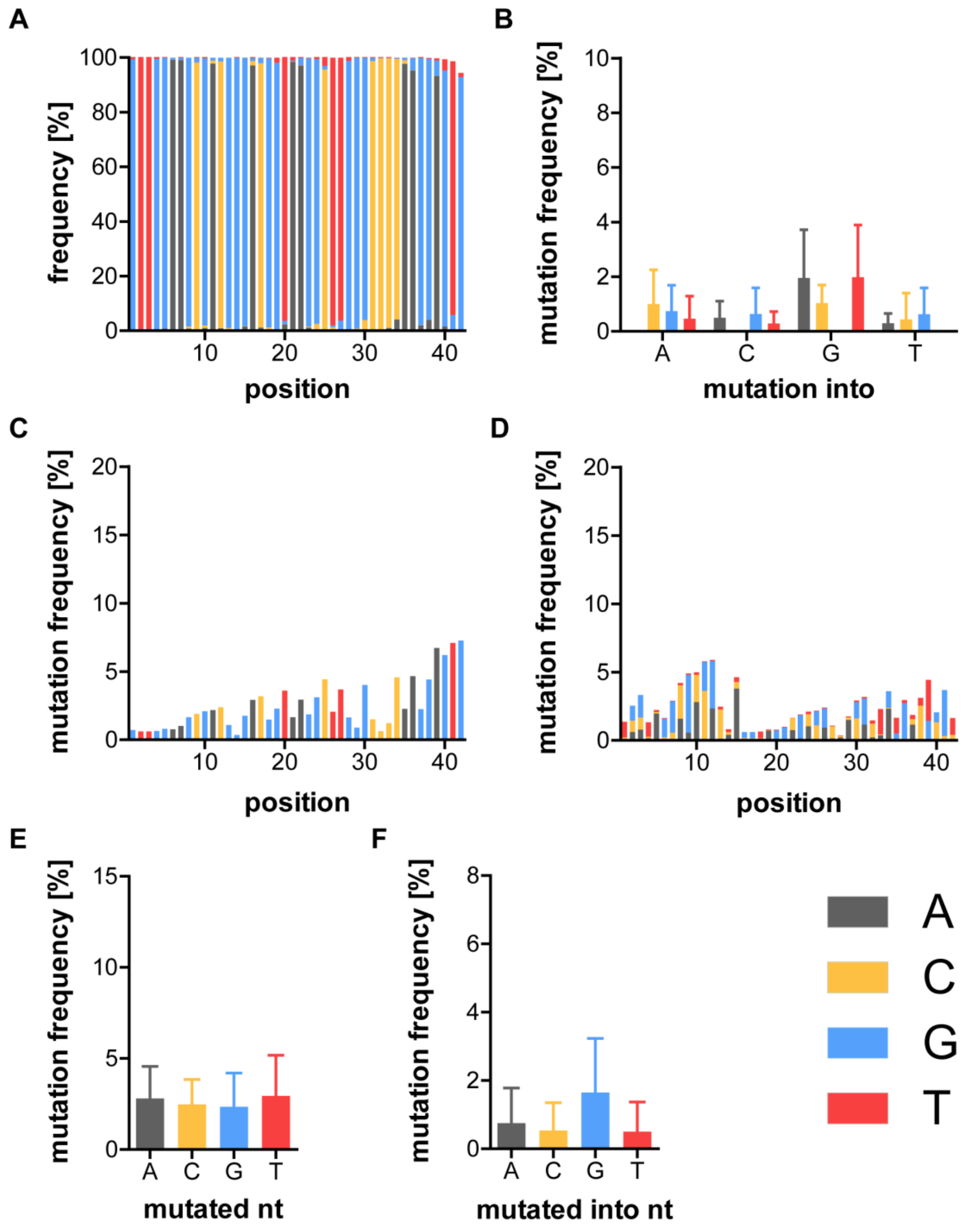

Figure S3: Mutation analysis of C12_T_w/o

(**A**) Frequency of the different nucleotides at all positions of the random region. (**B**) Frequencies with which one nucleotide (denoted by colour) mutates into another nucleotide (denoted on the x-axis). Depicted is the mean and SD. Mutation frequency at all positions of the random region of (**C**) the original nucleotide and (**D**) the nucleotide that has been mutated into. Mutation frequencies of the original nucleotides (**E**) and the nucleotides that has been mutated into (**F**). Depicted is the mean and SD.

Figure S4

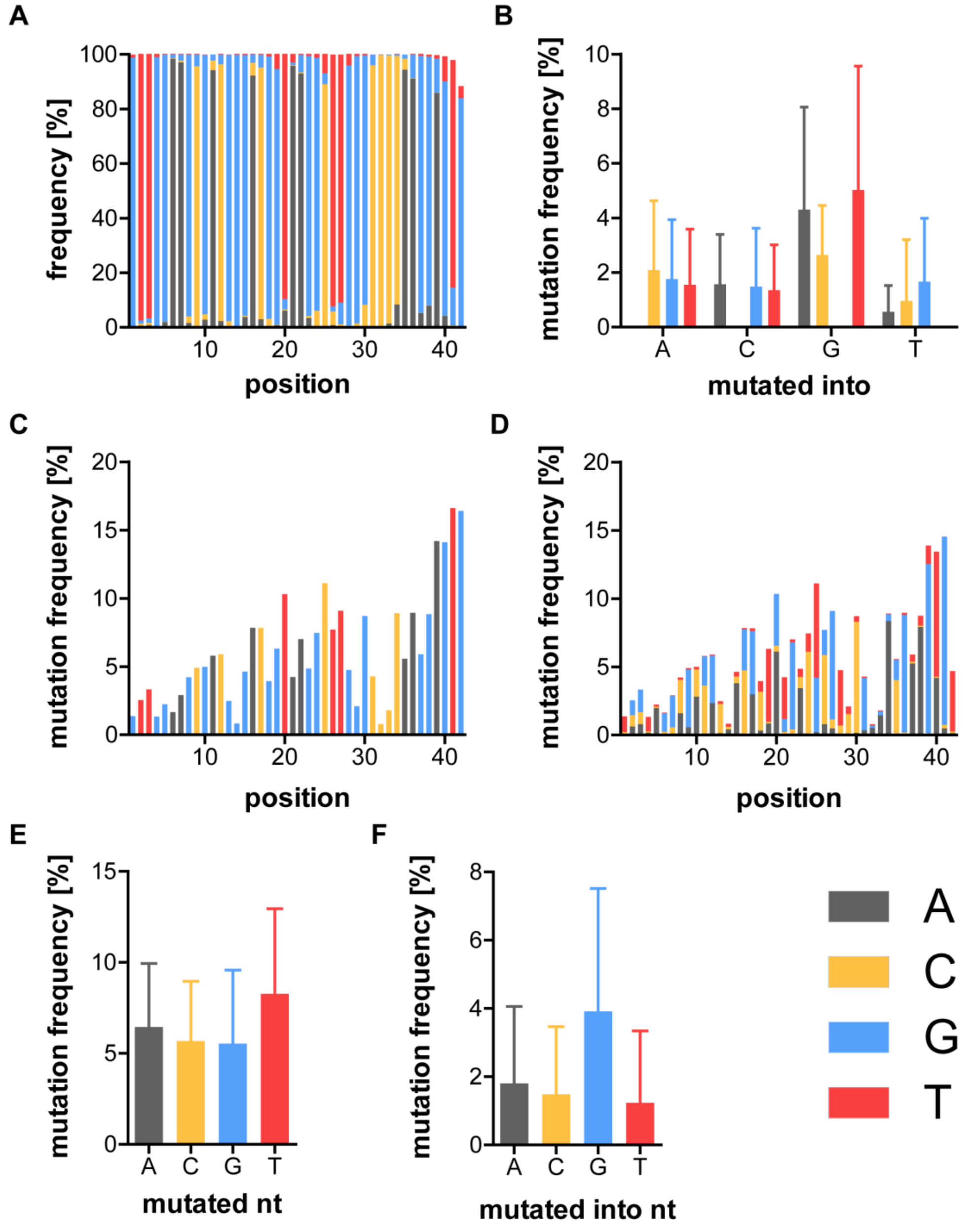

Figure S4: Mutation analysis of C12_EdU

(**A**) Frequency of the different nucleotides at all positions of the random region. (**B**) Frequencies with which one nucleotide (denoted by colour) mutates into another nucleotide (denoted on the x-axis). Depicted is the mean and SD. Mutation frequency at all positions of the random region of (**C**) the original nucleotide and (**D**) the nucleotide that has been mutated into. Mutation frequencies of the original nucleotides (**E**) and the nucleotides that has been mutated into (**F**). Depicted is the mean and SD.

Figure S5

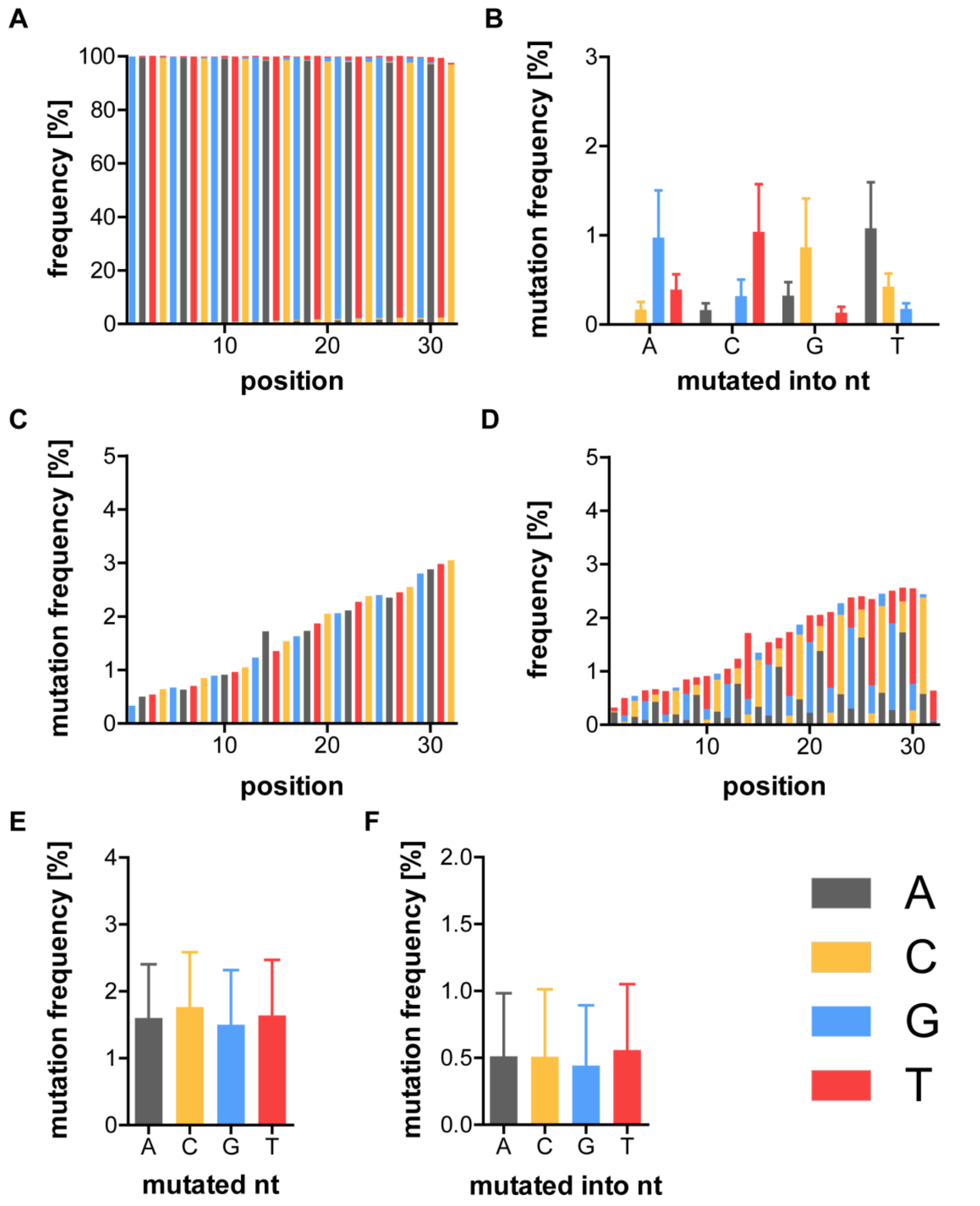

Figure S5: Mutation analysis of FT2_GATC

(**A**) Frequency of the different nucleotides at all positions of the random region. (**B**) Frequencies with which one nucleotide (denoted by colour) mutates into another nucleotide (denoted on the x-axis). Depicted is the mean and SD. Mutation frequency at all positions of the random region of (**C**) the original nucleotide and (**D**) the nucleotide that has been mutated into. Mutation frequencies of the original nucleotides (**E**) and the nucleotides that has been mutated into (**F**). Depicted is the mean and SD.

Figure S6

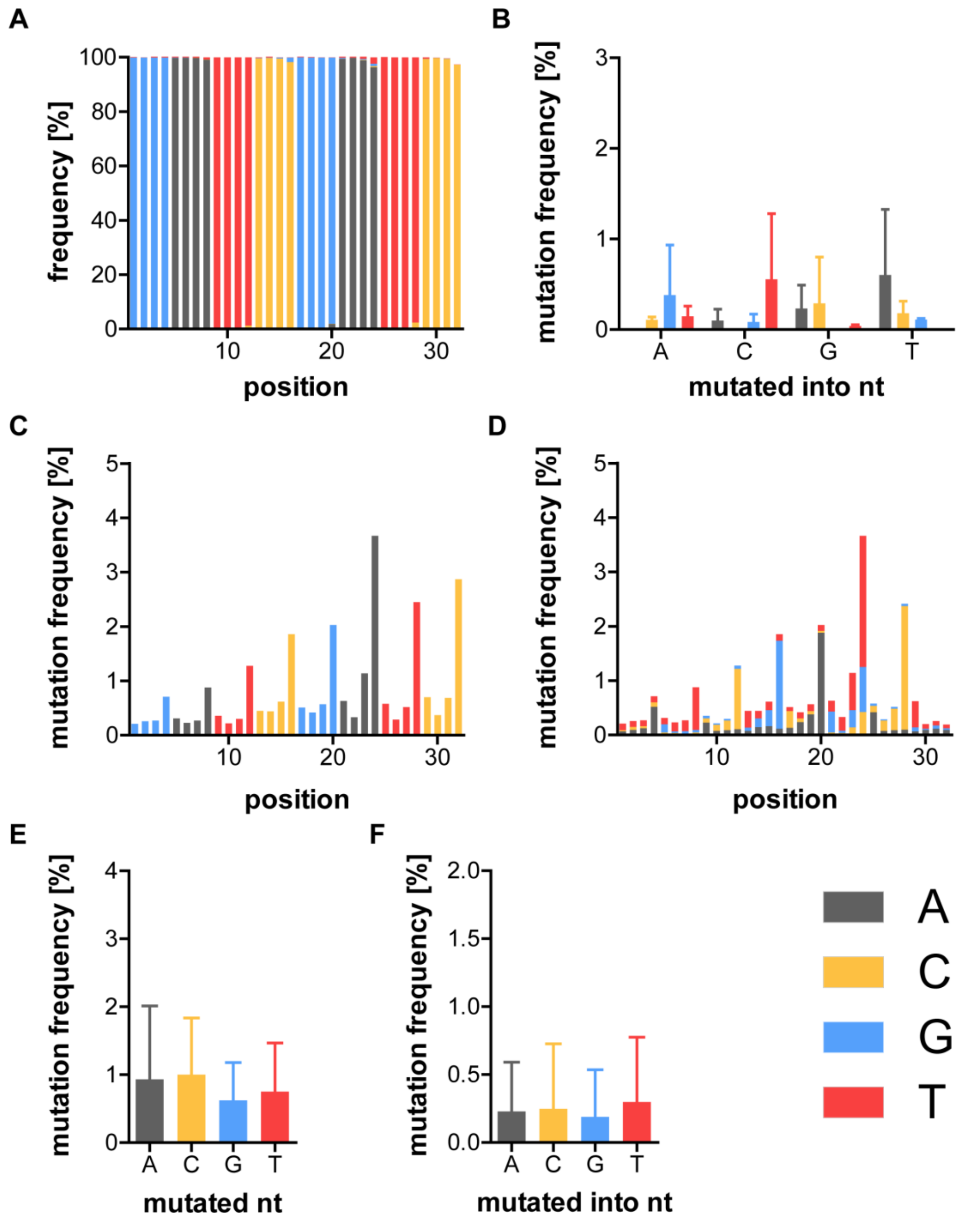

Figure S6: Mutation analysis of FT2_G4A4T4C4

(**A**) Frequency of the different nucleotides at all positions of the random region. (**B**) Frequencies with which one nucleotide (denoted by colour) mutates into another nucleotide (denoted on the x-axis). Depicted is the mean and SD. Mutation frequency at all positions of the random region of (**C**) the original nucleotide and (**D**) the nucleotide that has been mutated into. Mutation frequencies of the original nucleotides (**E**) and the nucleotides that has been mutated into (**F**). Depicted is the mean and SD.

Figure S7

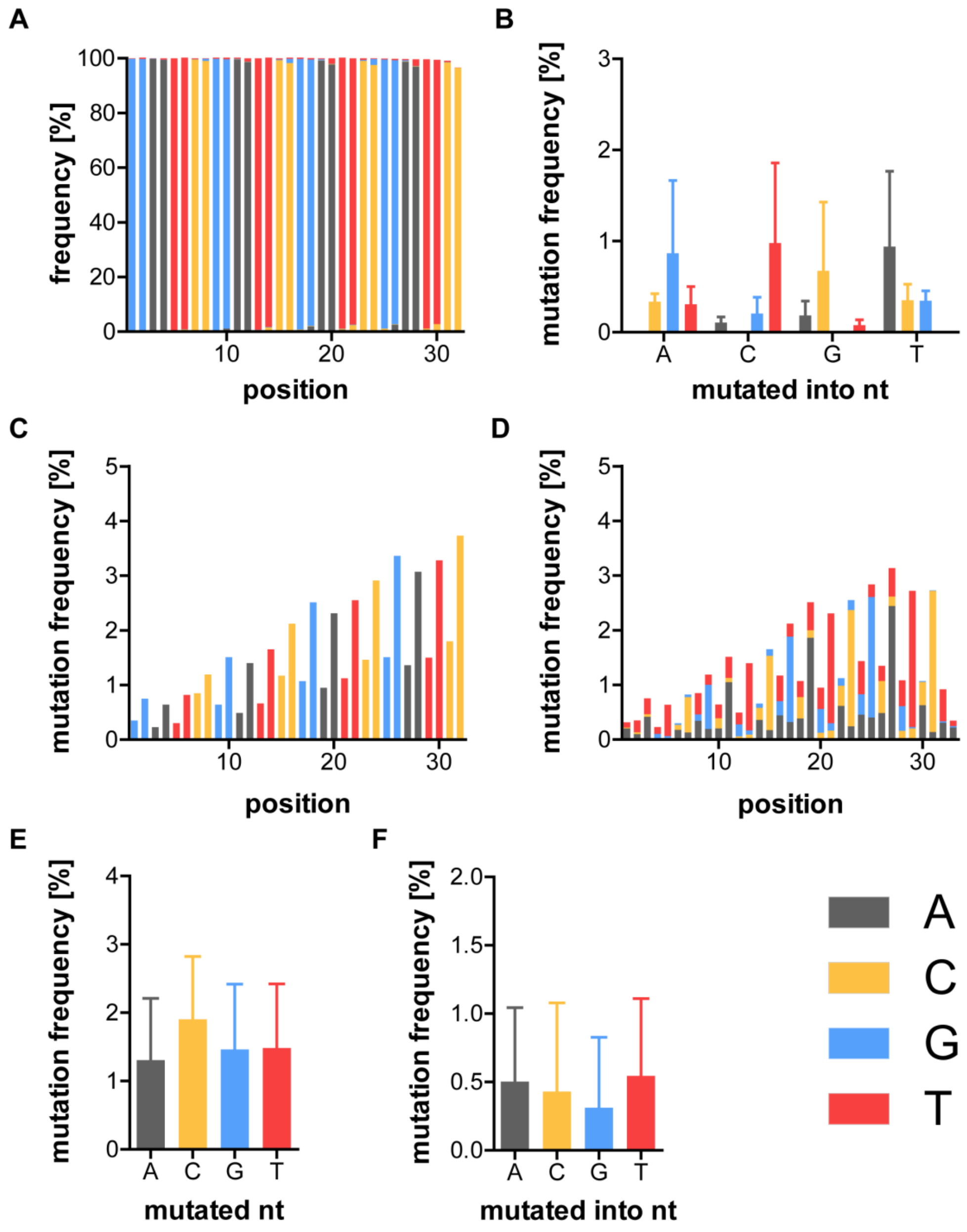

Figure S7: Mutation analysis of FT2_G2A2T2C2

(**A**) Frequency of the different nucleotides at all positions of the random region. (**B**) Frequencies with which one nucleotide (denoted by colour) mutates into another nucleotide (denoted on the x-axis). Depicted is the mean and SD. Mutation frequency at all positions of the random region of (**C**) the original nucleotide and (**D**) the nucleotide that has been mutated into. Mutation frequencies of the original nucleotides (**E**) and the nucleotides that has been mutated into (**F**). Depicted is the mean and SD.

Figure S8

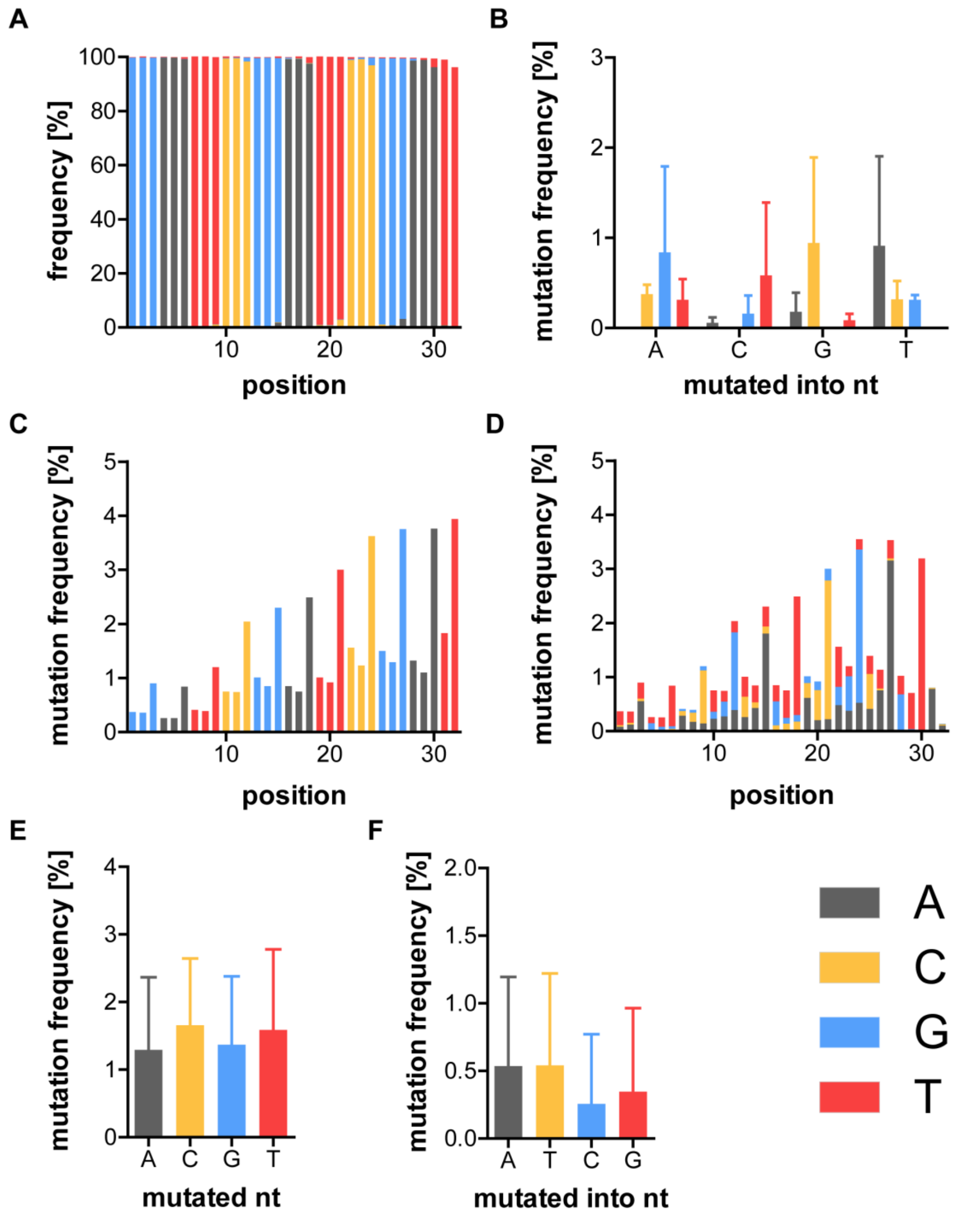

Figure S8: Mutation analysis of FT2_G3A3T3C3

(**A**) Frequency of the different nucleotides at all positions of the random region. (**B**) Frequencies with which one nucleotide (denoted by colour) mutates into another nucleotide (denoted on the x-axis). Depicted is the mean and SD. Mutation frequency at all positions of the random region of (**C**) the original nucleotide and (**D**) the nucleotide that has been mutated into. Mutation frequencies of the original nucleotides (**E**) and the nucleotides that has been mutated into (**F**). Depicted is the mean and SD.

Figure S9

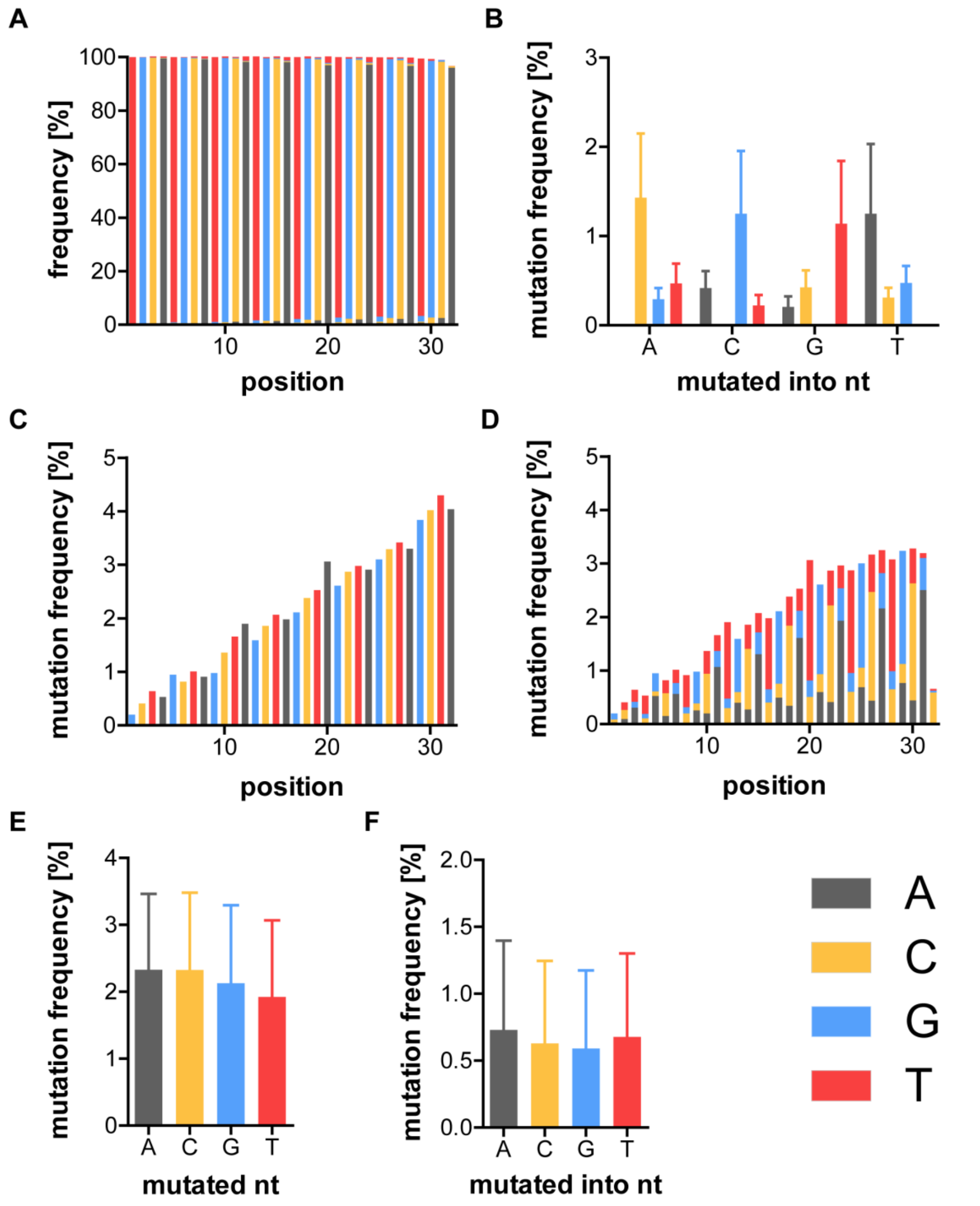

Figure S9: Mutation analysis of FT2_TGCA

(**A**) Frequency of the different nucleotides at all positions of the random region. (**B**) Frequencies with which one nucleotide (denoted by colour) mutates into another nucleotide (denoted on the x-axis). Depicted is the mean and SD. Mutation frequency at all positions of the random region of (**C**) the original nucleotide and (**D**) the nucleotide that has been mutated into. Mutation frequencies of the original nucleotides (**E**) and the nucleotides that has been mutated into (**F**). Depicted is the mean and SD.

Figure S10

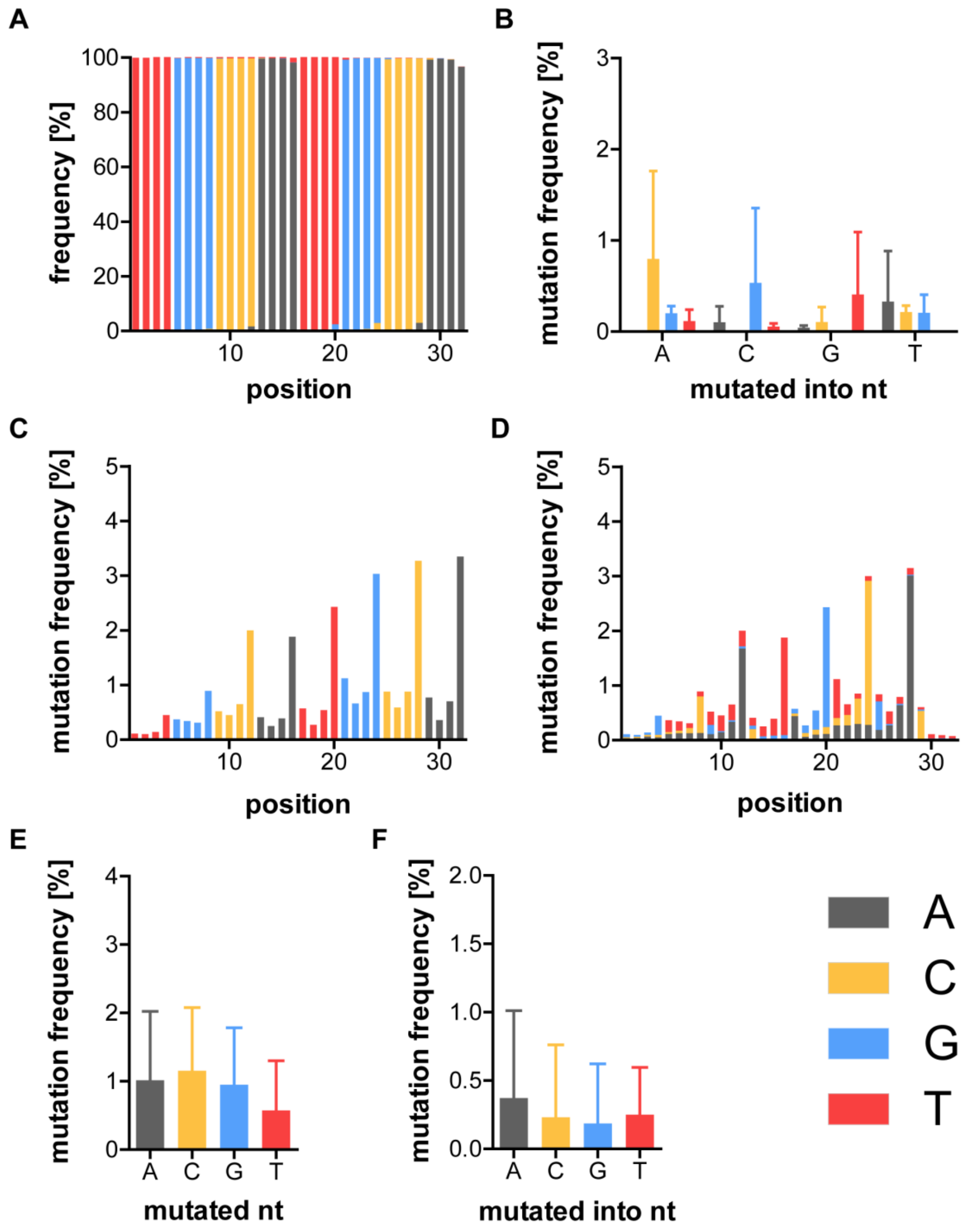

Figure S10: Mutation analysis of FT2_T4G4C4A4

(**A**) Frequency of the different nucleotides at all positions of the random region. (**B**) Frequencies with which one nucleotide (denoted by colour) mutates into another nucleotide (denoted on the x-axis). Depicted is the mean and SD. Mutation frequency at all positions of the random region of (**C**) the original nucleotide and (**D**) the nucleotide that has been mutated into. Mutation frequencies of the original nucleotides (**E**) and the nucleotides that has been mutated into (**F**). Depicted is the mean and SD.

Figure S11

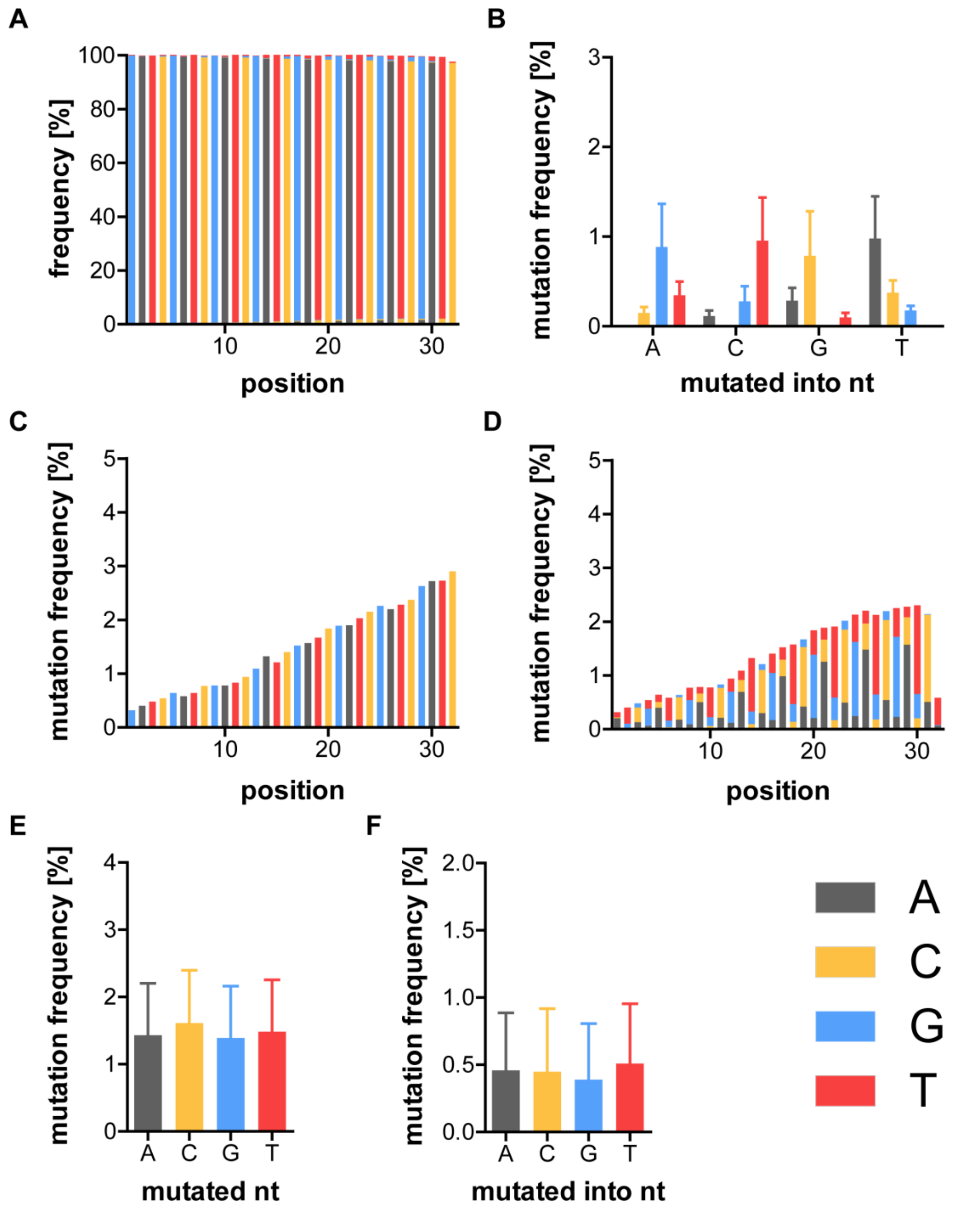

Figure S11: Mutation analysis of FT2_GATC_II

(**A**) Frequency of the different nucleotides at all positions of the random region. (**B**) Frequencies with which one nucleotide (denoted by colour) mutates into another nucleotide (denoted on the x-axis). Depicted is the mean and SD. Mutation frequency at all positions of the random region of (**C**) the original nucleotide and (**D**) the nucleotide that has been mutated into. Mutation frequencies of the original nucleotides (**E**) and the nucleotides that has been mutated into (**F**). Depicted is the mean and SD.

Figure S12

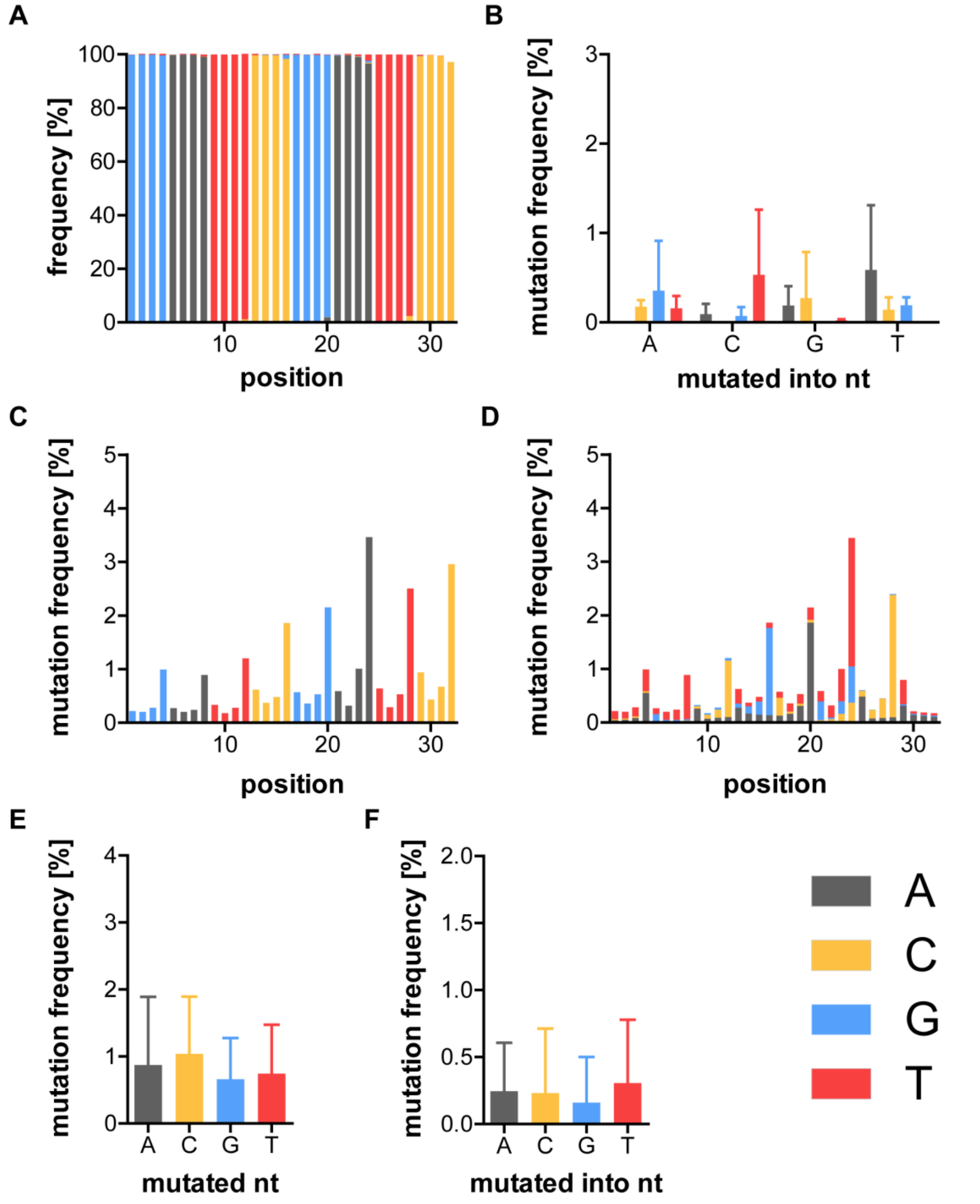

Figure S12: Mutation analysis of FT2_G4A4T4C4_II

(**A**) Frequency of the different nucleotides at all positions of the random region. (**B**) Frequencies with which one nucleotide (denoted by colour) mutates into another nucleotide (denoted on the x-axis). Depicted is the mean and SD. Mutation frequency at all positions of the random region of (**C**) the original nucleotide and (**D**) the nucleotide that has been mutated into. Mutation frequencies of the original nucleotides (**E**) and the nucleotides that has been mutated into (**F**). Depicted is the mean and SD.

Figure S13

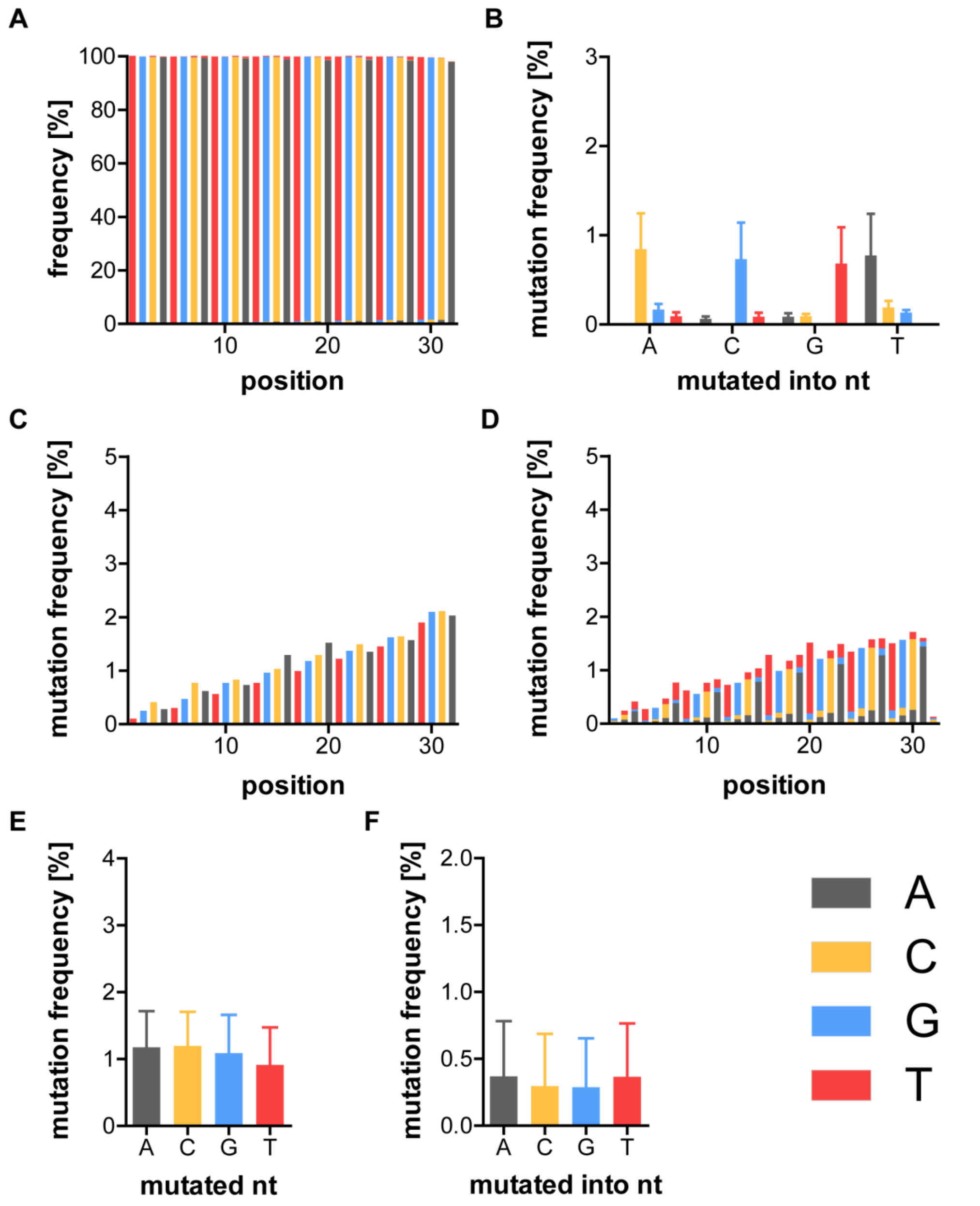

Figure S13: Mutation analysis of D3_TGCA

(**A**) Frequency of the different nucleotides at all positions of the random region. (**B**) Frequencies with which one nucleotide (denoted by colour) mutates into another nucleotide (denoted on the x-axis). Depicted is the mean and SD. Mutation frequency at all positions of the random region of (**C**) the original nucleotide and (**D**) the nucleotide that has been mutated into. Mutation frequencies of the original nucleotides (**E**) and the nucleotides that has been mutated into (**F**). Depicted is the mean and SD.

Figure S14

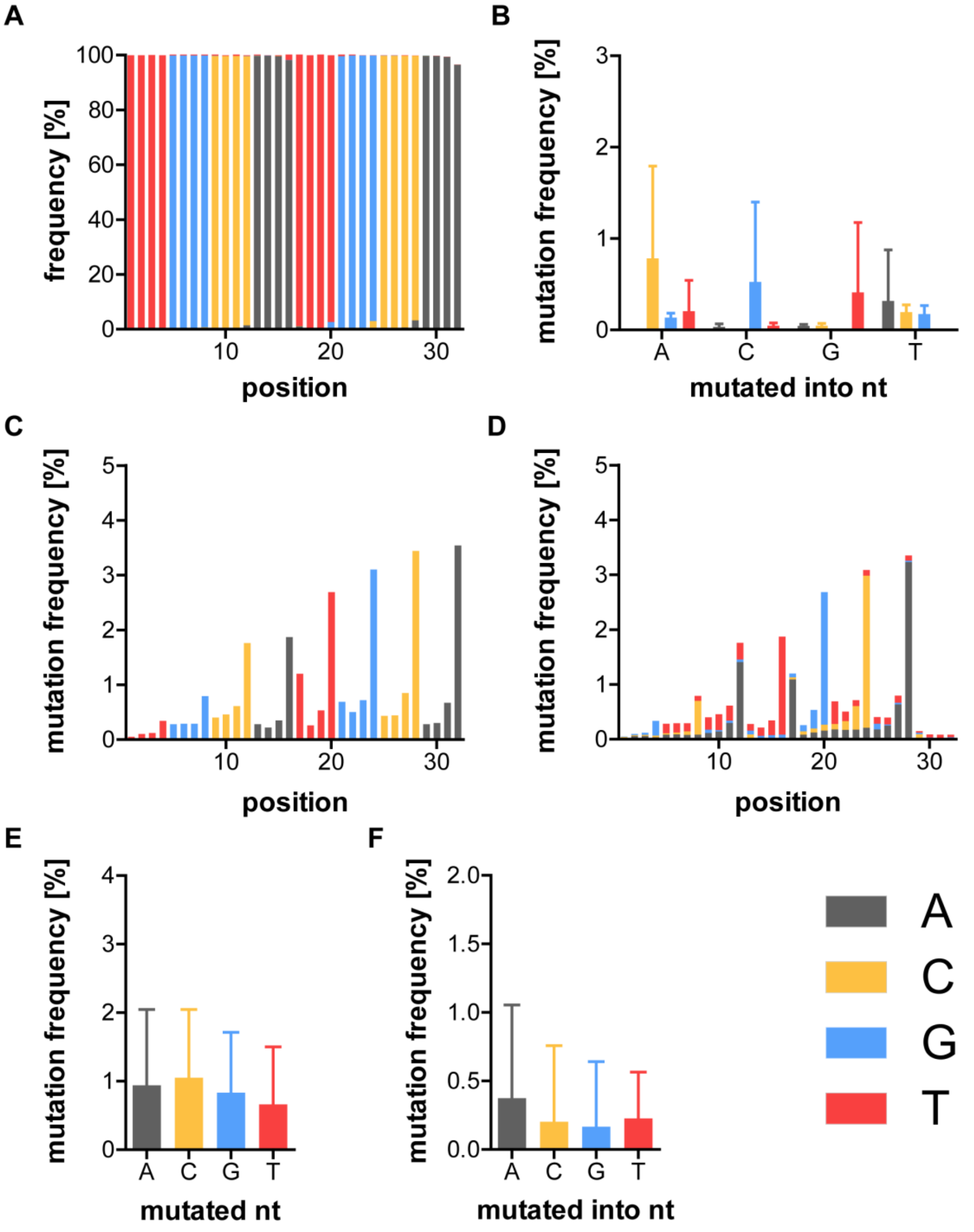

Figure S14: Mutation analysis of D3_T4G4C4A4

(**A**) Frequency of the different nucleotides at all positions of the random region. (**B**) Frequencies with which one nucleotide (denoted by colour) mutates into another nucleotide (denoted on the x-axis). Depicted is the mean and SD. Mutation frequency at all positions of the random region of (**C**) the original nucleotide and (**D**) the nucleotide that has been mutated into. Mutation frequencies of the original nucleotides (**E**) and the nucleotides that has been mutated into (**F**). Depicted is the mean and SD.

Figure S15

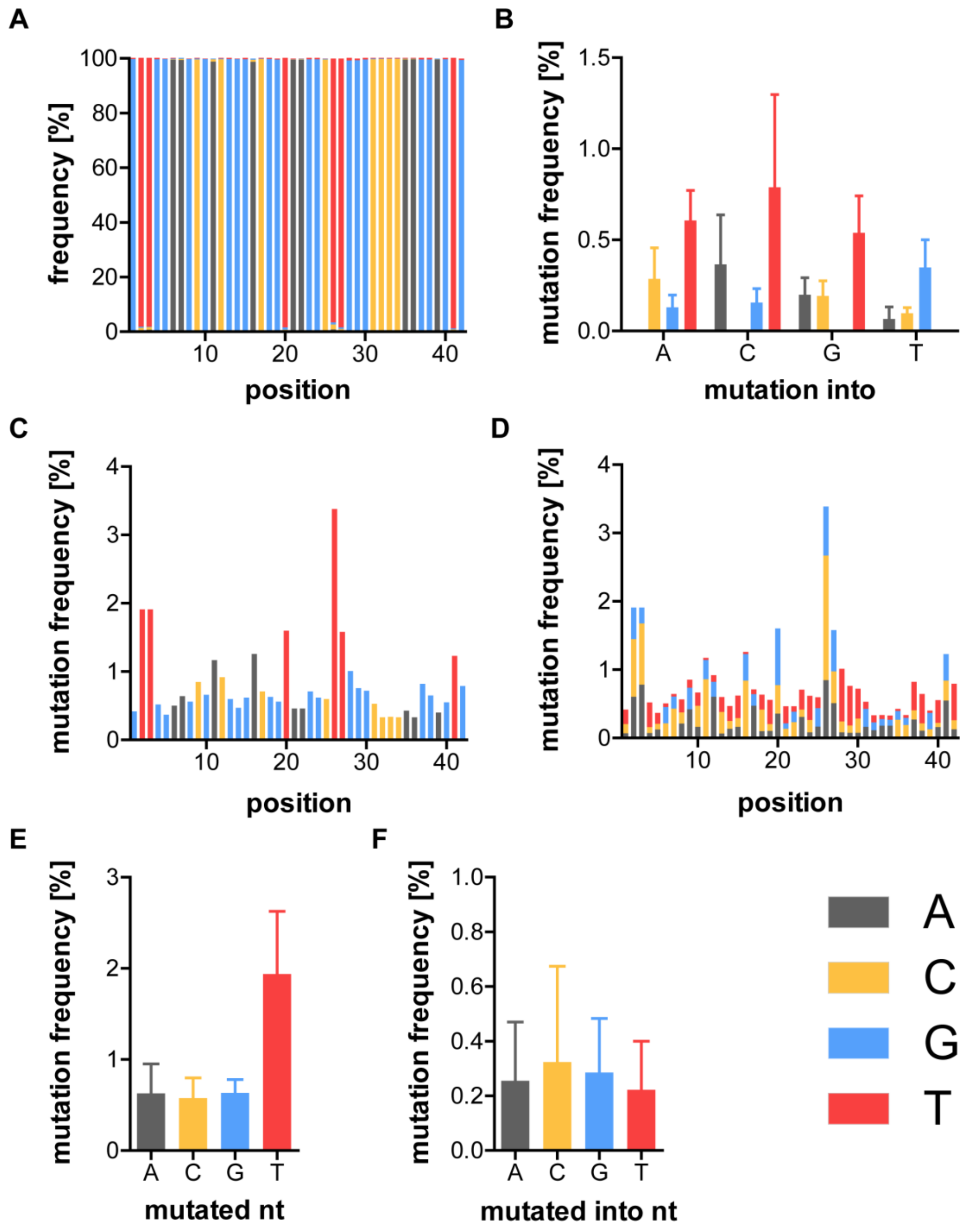

Figure S15: Mutation analysis of C12_EdU after omission of shortened sequences

(**A**) Frequency of the different nucleotides at all positions of the random region. (**B**) Frequencies with which one nucleotide (denoted by colour) mutates into another nucleotide (denoted on the x-axis). Depicted is the mean and SD. Mutation frequency at all positions of the random region of (**C**) the original nucleotide and (**D**) the nucleotide that has been mutated into. Mutation frequencies of the original nucleotides (**E**) and the nucleotides that has been mutated into (**F**). Depicted is the mean and SD.

Figure S16

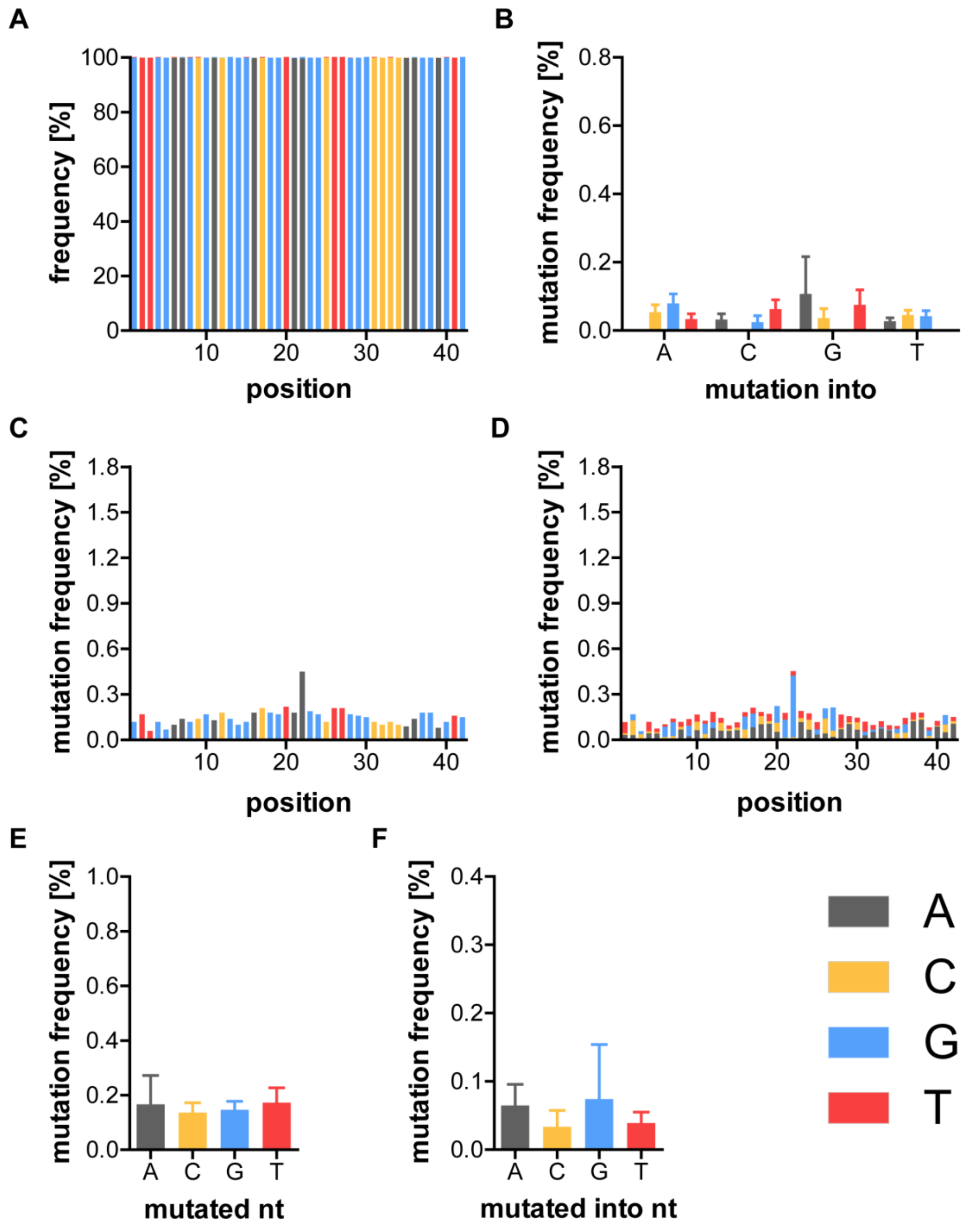

Figure S16: Mutation analysis of C12_T_PWO after omission of shortened sequences

(**A**) Frequency of the different nucleotides at all positions of the random region. (**B**) Frequencies with which one nucleotide (denoted by colour) mutates into another nucleotide (denoted on the x-axis). Depicted is the mean and SD. Mutation frequency at all positions of the random region of (**C**) the original nucleotide and (**D**) the nucleotide that has been mutated into. Mutation frequencies of the original nucleotides (**E**) and the nucleotides that has been mutated into (**F**). Depicted is the mean and SD.

Figure S17

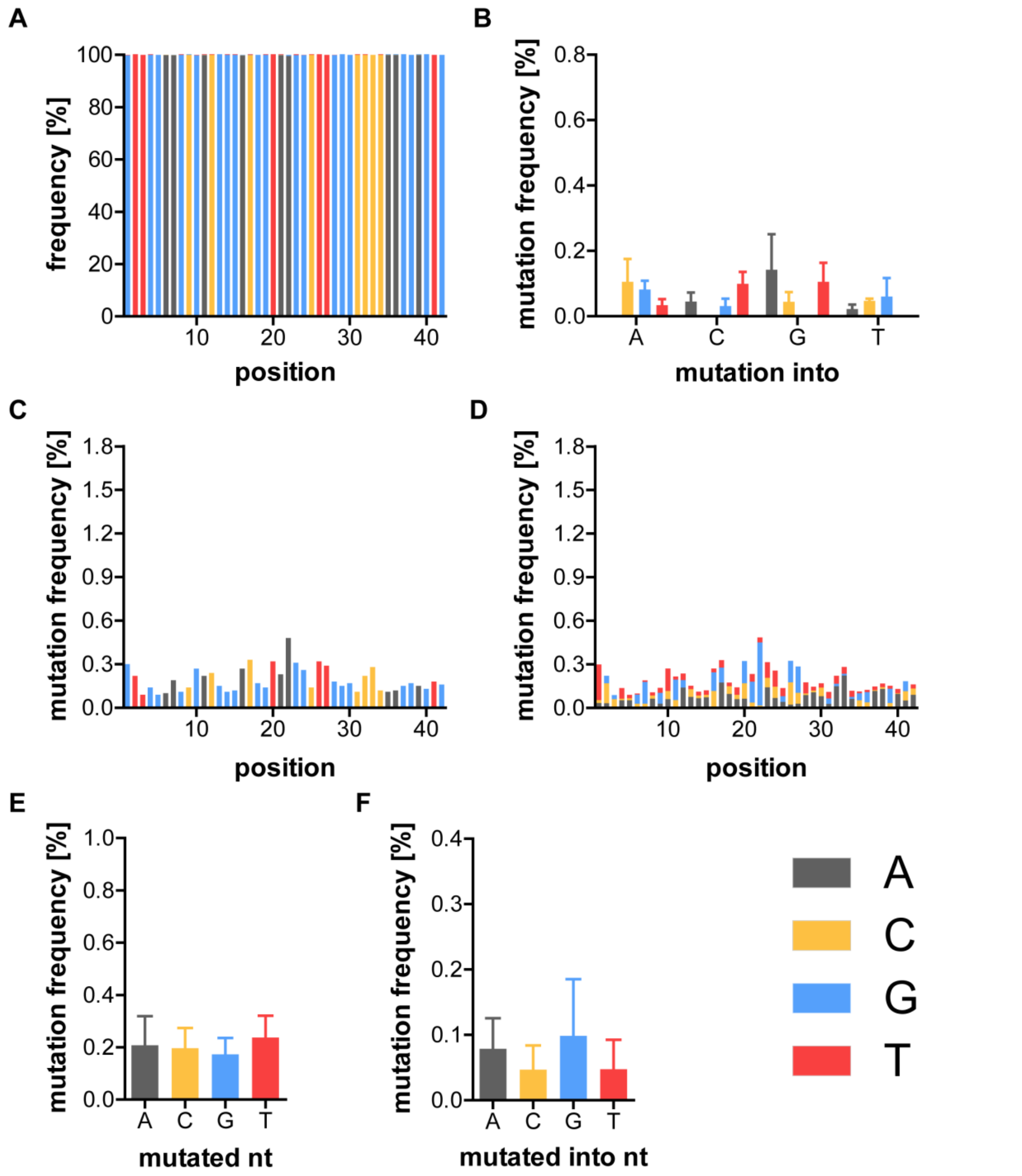

Figure S17: Mutation analysis of C12_T_PWO after omission of shortened sequences

(**A**) Frequency of the different nucleotides at all positions of the random region. (**B**) Frequencies with which one nucleotide (denoted by colour) mutates into another nucleotide (denoted on the x-axis). Depicted is the mean and SD. Mutation frequency at all positions of the random region of (**C**) the original nucleotide and (**D**) the nucleotide that has been mutated into. Mutation frequencies of the original nucleotides (**E**) and the nucleotides that has been mutated into (**F**). Depicted is the mean and SD.

Figure S18

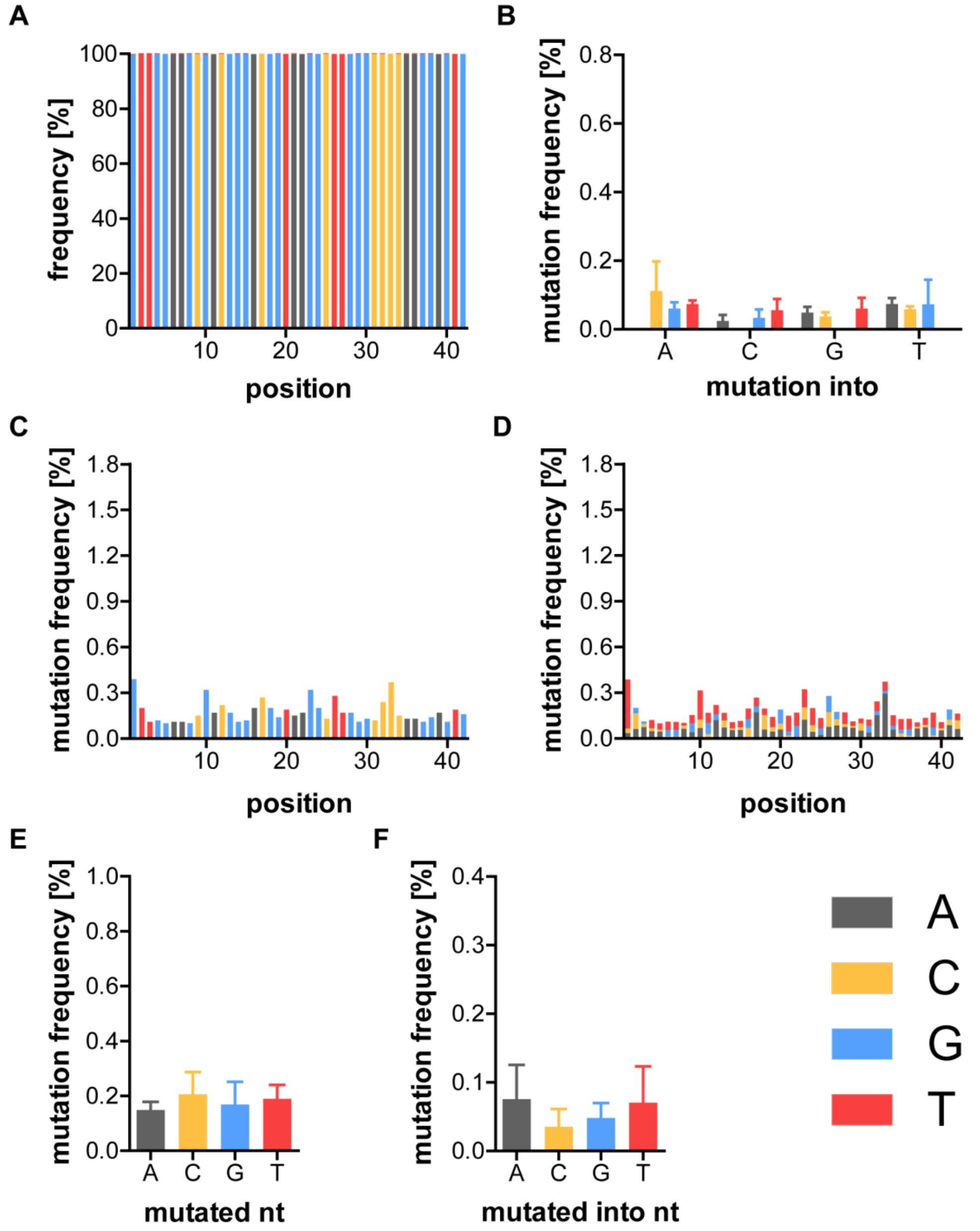

Figure S18: Mutation analysis of C12_T_w/o after omission of shortened sequences

(**A**) Frequency of the different nucleotides at all positions of the random region. (**B**) Frequencies with which one nucleotide (denoted by colour) mutates into another nucleotide (denoted on the x-axis). Depicted is the mean and SD. Mutation frequency at all positions of the random region of (**C**) the original nucleotide and (**D**) the nucleotide that has been mutated into. Mutation frequencies of the original nucleotides (**E**) and the nucleotides that has been mutated into (**F**). Depicted is the mean and SD.

Figure S19

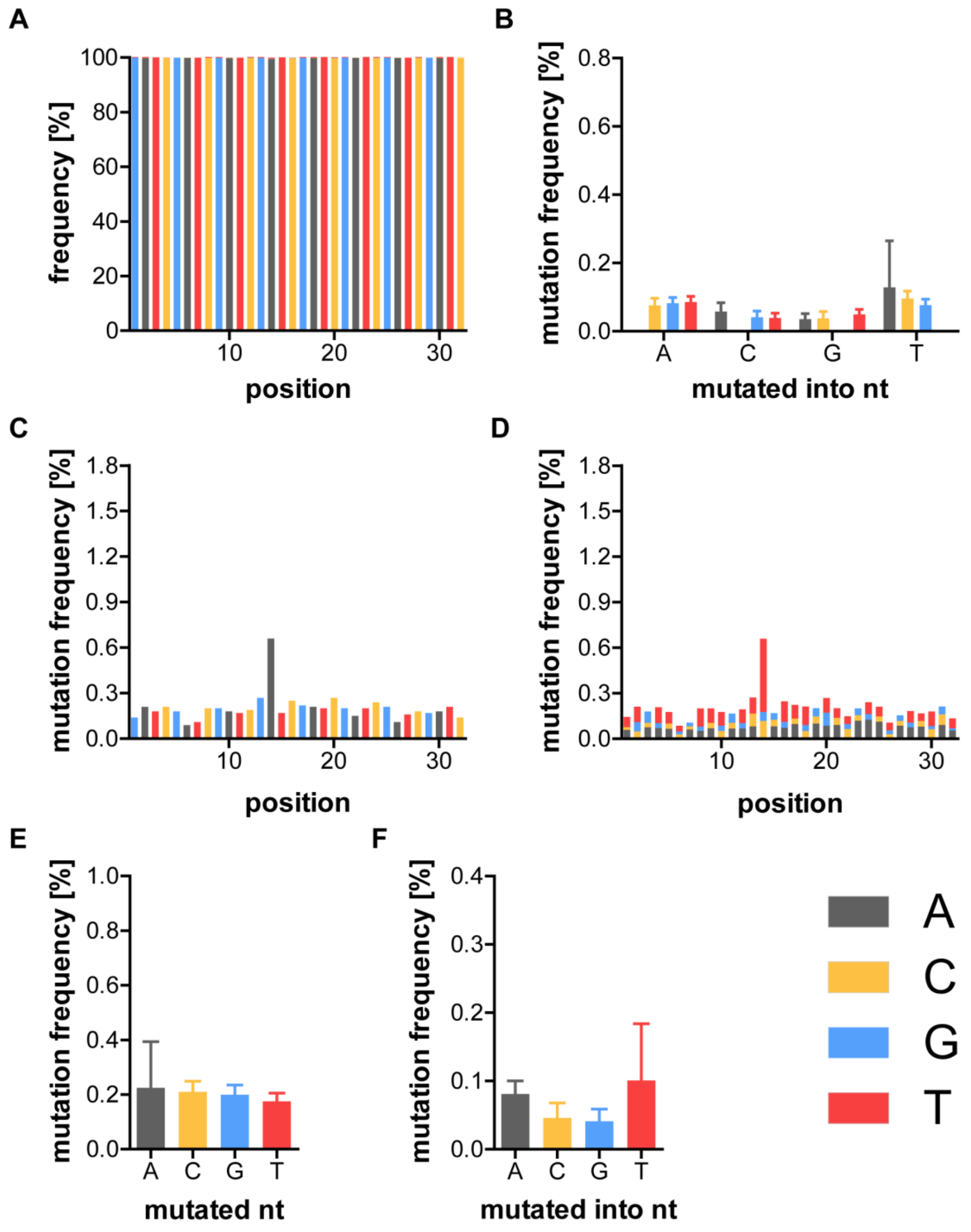

Figure S19: Mutation analysis of FT2_GATC after omission of shortened sequences

(**A**) Frequency of the different nucleotides at all positions of the random region. (**B**) Frequencies with which one nucleotide (denoted by colour) mutates into another nucleotide (denoted on the x-axis). Depicted is the mean and SD. Mutation frequency at all positions of the random region of (**C**) the original nucleotide and (**D**) the nucleotide that has been mutated into. Mutation frequencies of the original nucleotides (**E**) and the nucleotides that has been mutated into (**F**). Depicted is the mean and SD.

Figure S20

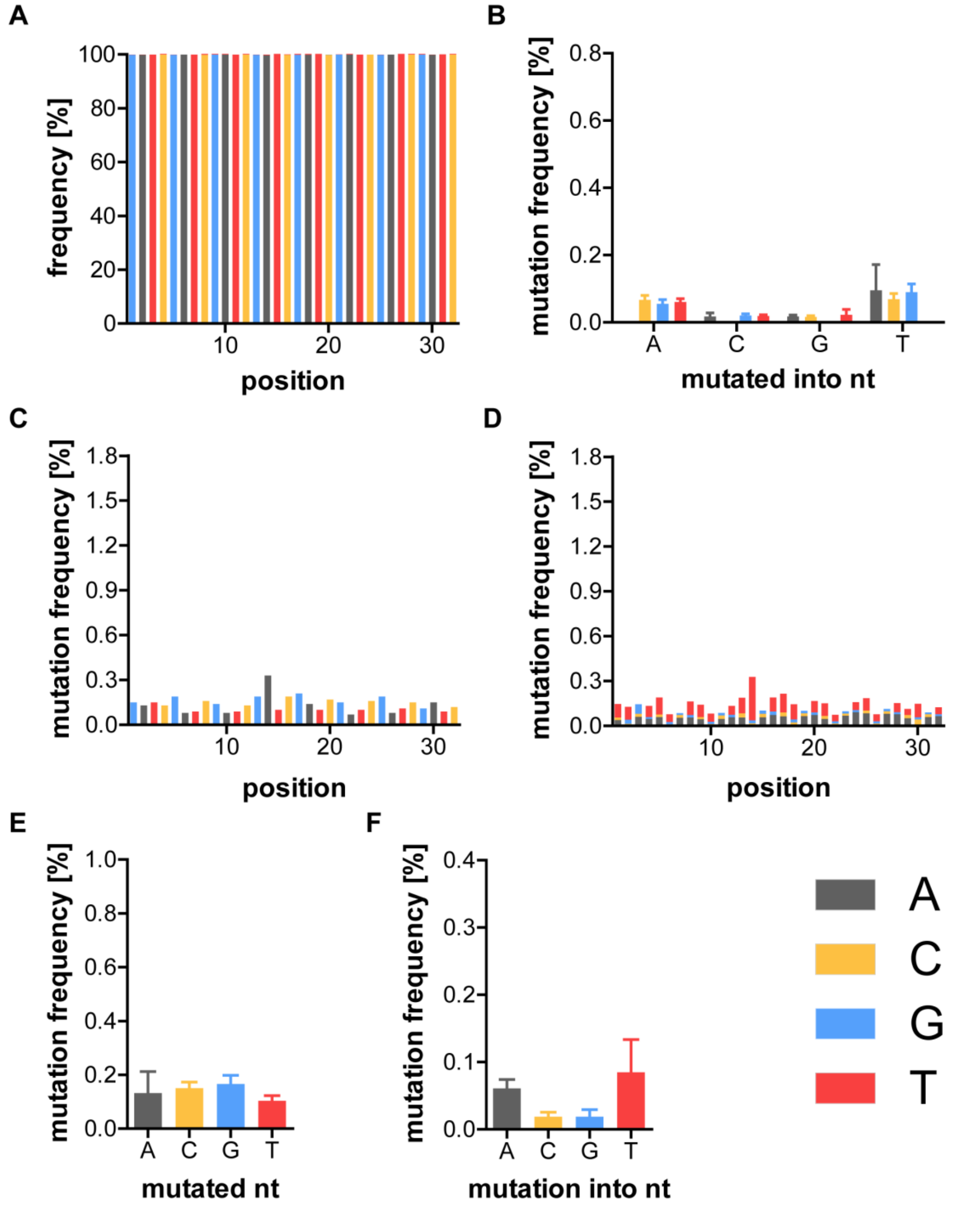

Figure S20: Mutation analysis of FT2_GATC_II after omission of shortened sequences

(**A**) Frequency of the different nucleotides at all positions of the random region. (**B**) Frequencies with which one nucleotide (denoted by colour) mutates into another nucleotide (denoted on the x-axis). Depicted is the mean and SD. Mutation frequency at all positions of the random region of (**C**) the original nucleotide and (**D**) the nucleotide that has been mutated into. Mutation frequencies of the original nucleotides (**E**) and the nucleotides that has been mutated into (**F**). Depicted is the mean and SD.

Figure S21

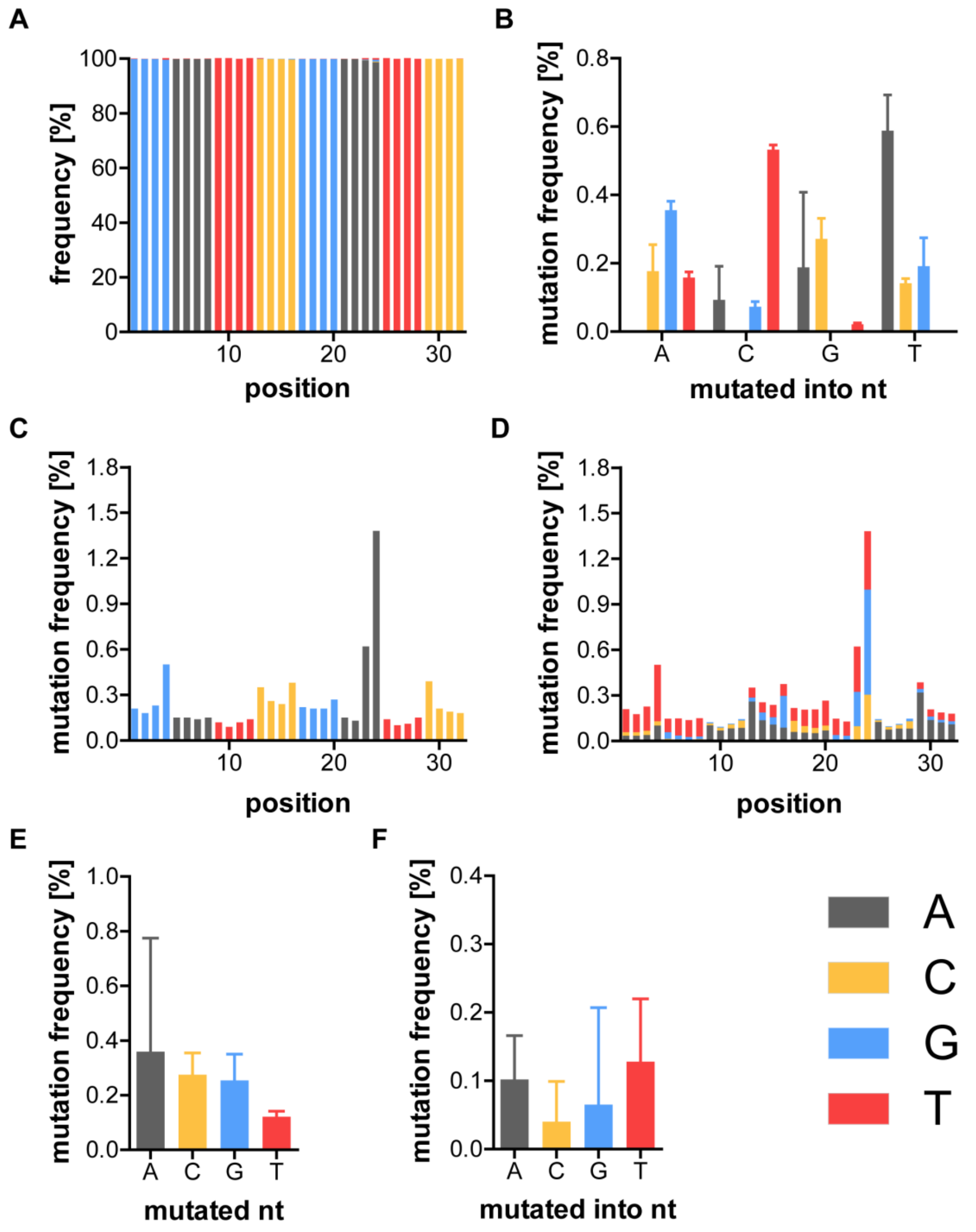

Figure S21: Mutation analysis of FT2_G4A4T4C4 after omission of shortened sequences

(**A**) Frequency of the different nucleotides at all positions of the random region. (**B**) Frequencies with which one nucleotide (denoted by colour) mutates into another nucleotide (denoted on the x-axis). Depicted is the mean and SD. Mutation frequency at all positions of the random region of (**C**) the original nucleotide and (**D**) the nucleotide that has been mutated into. Mutation frequencies of the original nucleotides (**E**) and the nucleotides that has been mutated into (**F**). Depicted is the mean and SD.

Figure S22

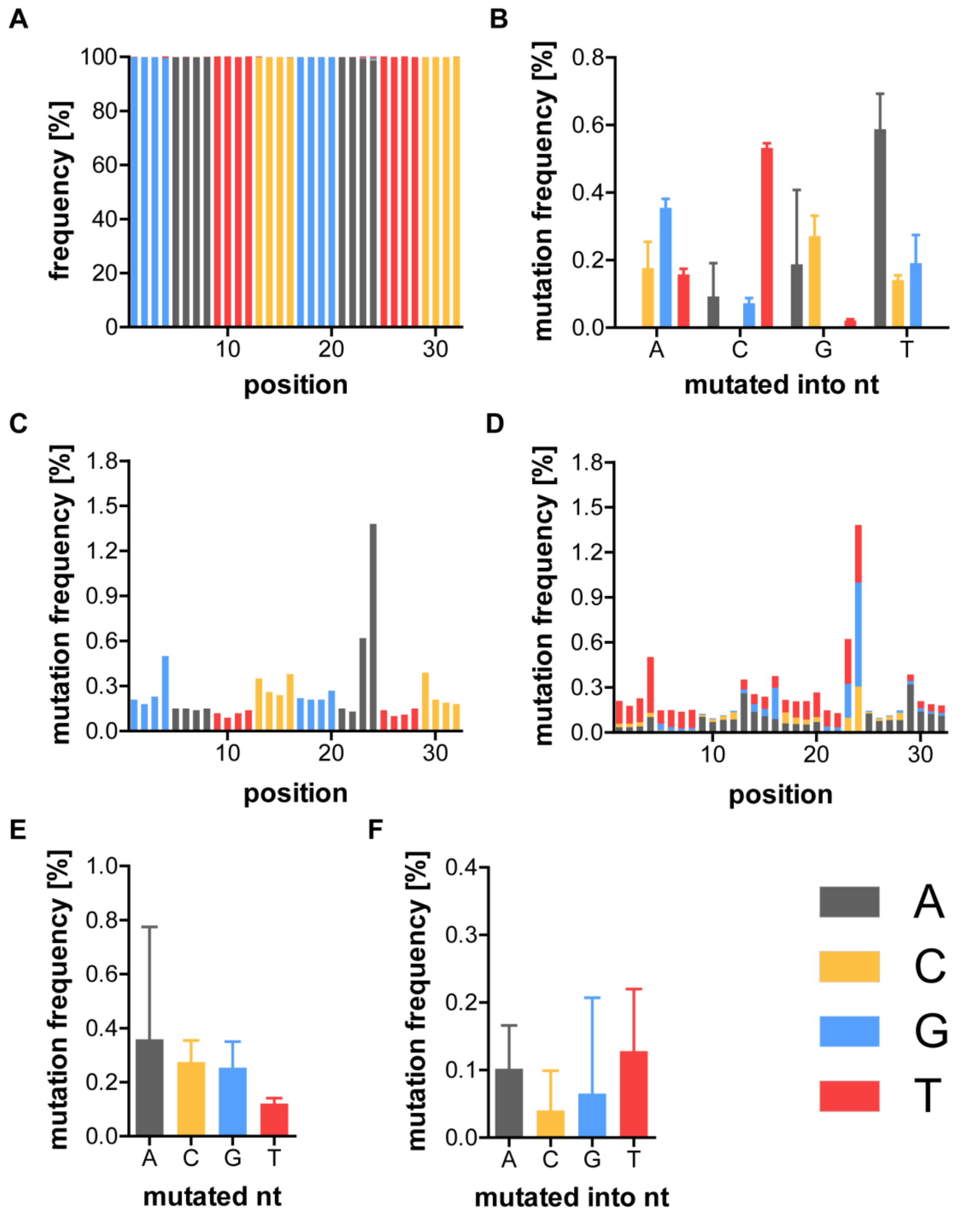

Figure S22: Mutation analysis of FT2_G4A4T4C4_II after omission of shortened sequences

(**A**) Frequency of the different nucleotides at all positions of the random region. (**B**) Frequencies with which one nucleotide (denoted by colour) mutates into another nucleotide (denoted on the x-axis). Depicted is the mean and SD. Mutation frequency at all positions of the random region of (**C**) the original nucleotide and (**D**) the nucleotide that has been mutated into. Mutation frequencies of the original nucleotides (**E**) and the nucleotides that has been mutated into (**F**). Depicted is the mean and SD.

Figure S23

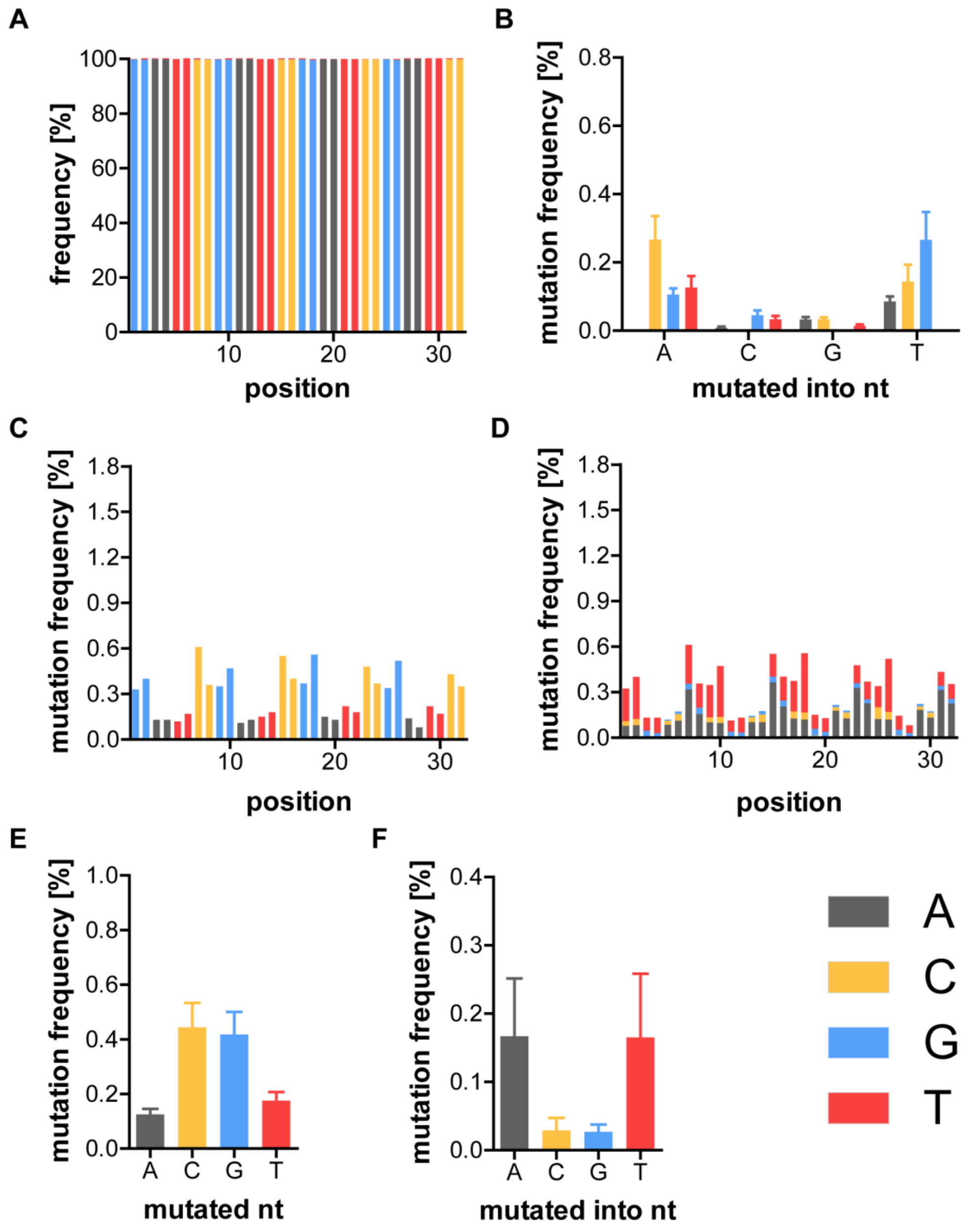

Figure S23: Mutation analysis of FT2_G2A2T2C2 after omission of shortened sequences

(**A**) Frequency of the different nucleotides at all positions of the random region. (**B**) Frequencies with which one nucleotide (denoted by colour) mutates into another nucleotide (denoted on the x-axis). Depicted is the mean and SD. Mutation frequency at all positions of the random region of (**C**) the original nucleotide and (**D**) the nucleotide that has been mutated into. Mutation frequencies of the original nucleotides (**E**) and the nucleotides that has been mutated into (**F**). Depicted is the mean and SD.

Figure S24

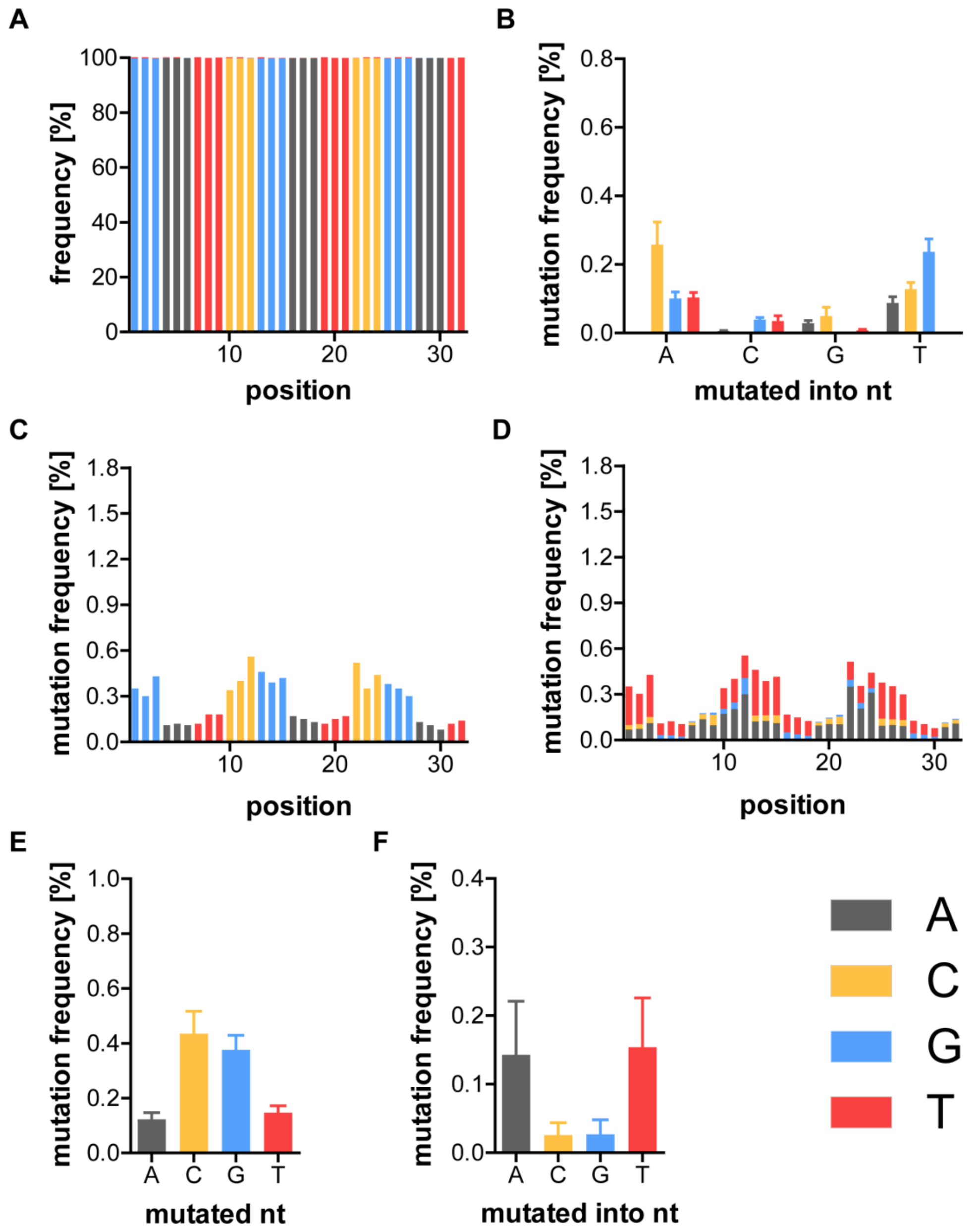

Figure S24: Mutation analysis of FT2_G3A3T3C3 after omission of shortened sequences

(**A**) Frequency of the different nucleotides at all positions of the random region. (**B**) Frequencies with which one nucleotide (denoted by colour) mutates into another nucleotide (denoted on the x-axis). Depicted is the mean and SD. Mutation frequency at all positions of the random region of (**C**) the original nucleotide and (**D**) the nucleotide that has been mutated into. Mutation frequencies of the original nucleotides (**E**) and the nucleotides that has been mutated into (**F**). Depicted is the mean and SD.

Figure S25

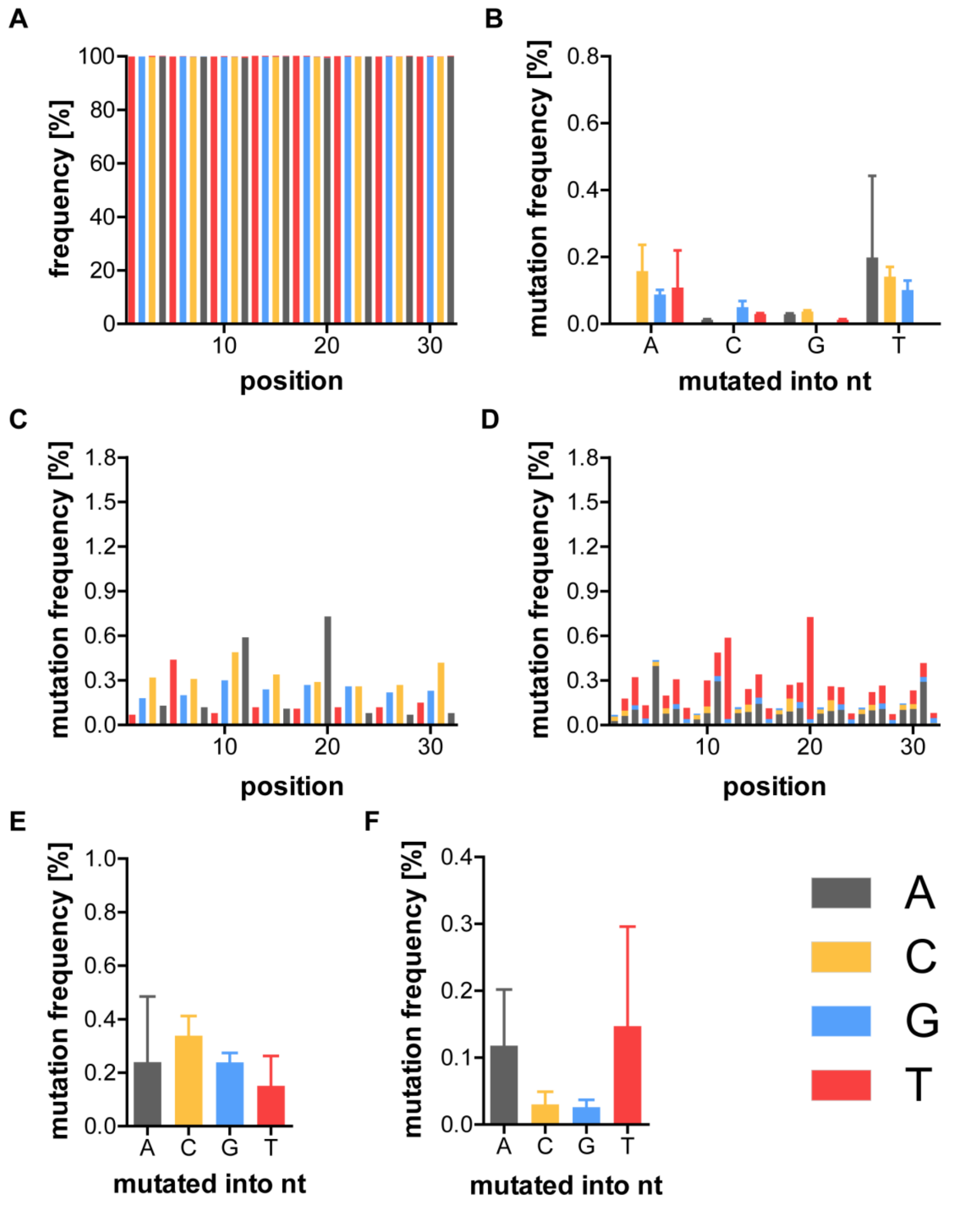

Figure S25: Mutation analysis of FT2_TGCA after omission of shortened sequences

(**A**) Frequency of the different nucleotides at all positions of the random region. (**B**) Frequencies with which one nucleotide (denoted by colour) mutates into another nucleotide (denoted on the x-axis). Depicted is the mean and SD. Mutation frequency at all positions of the random region of (**C**) the original nucleotide and (**D**) the nucleotide that has been mutated into. Mutation frequencies of the original nucleotides (**E**) and the nucleotides that has been mutated into (**F**). Depicted is the mean and SD.

Figure S26

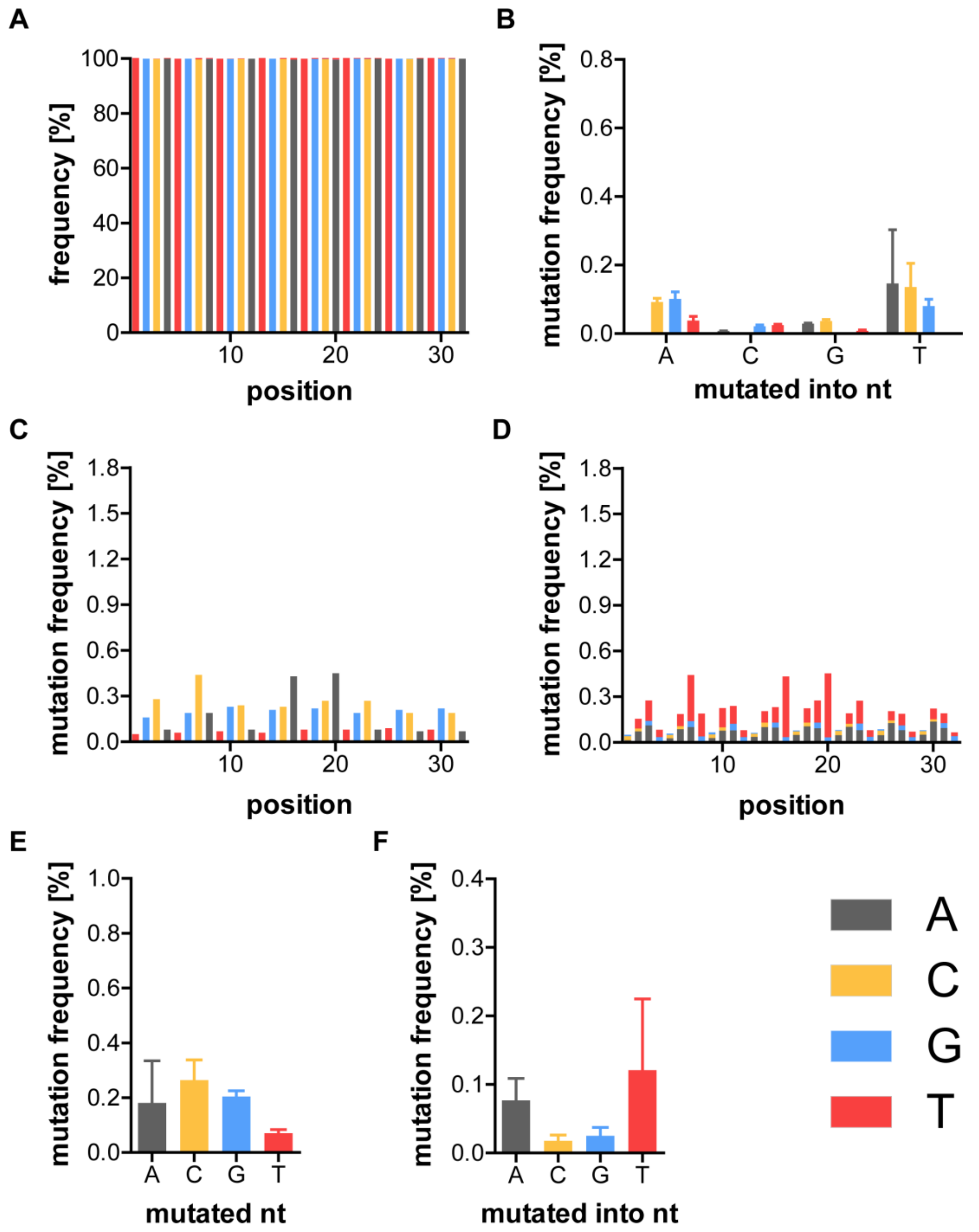

Figure S26: Mutation analysis of D3-TGCA after omission of shortened sequences

(**A**) Frequency of the different nucleotides at all positions of the random region. (**B**) Frequencies with which one nucleotide (denoted by colour) mutates into another nucleotide (denoted on the x-axis). Depicted is the mean and SD. Mutation frequency at all positions of the random region of (**C**) the original nucleotide and (**D**) the nucleotide that has been mutated into. Mutation frequencies of the original nucleotides (**E**) and the nucleotides that has been mutated into (**F**). Depicted is the mean and SD.

Figure S27

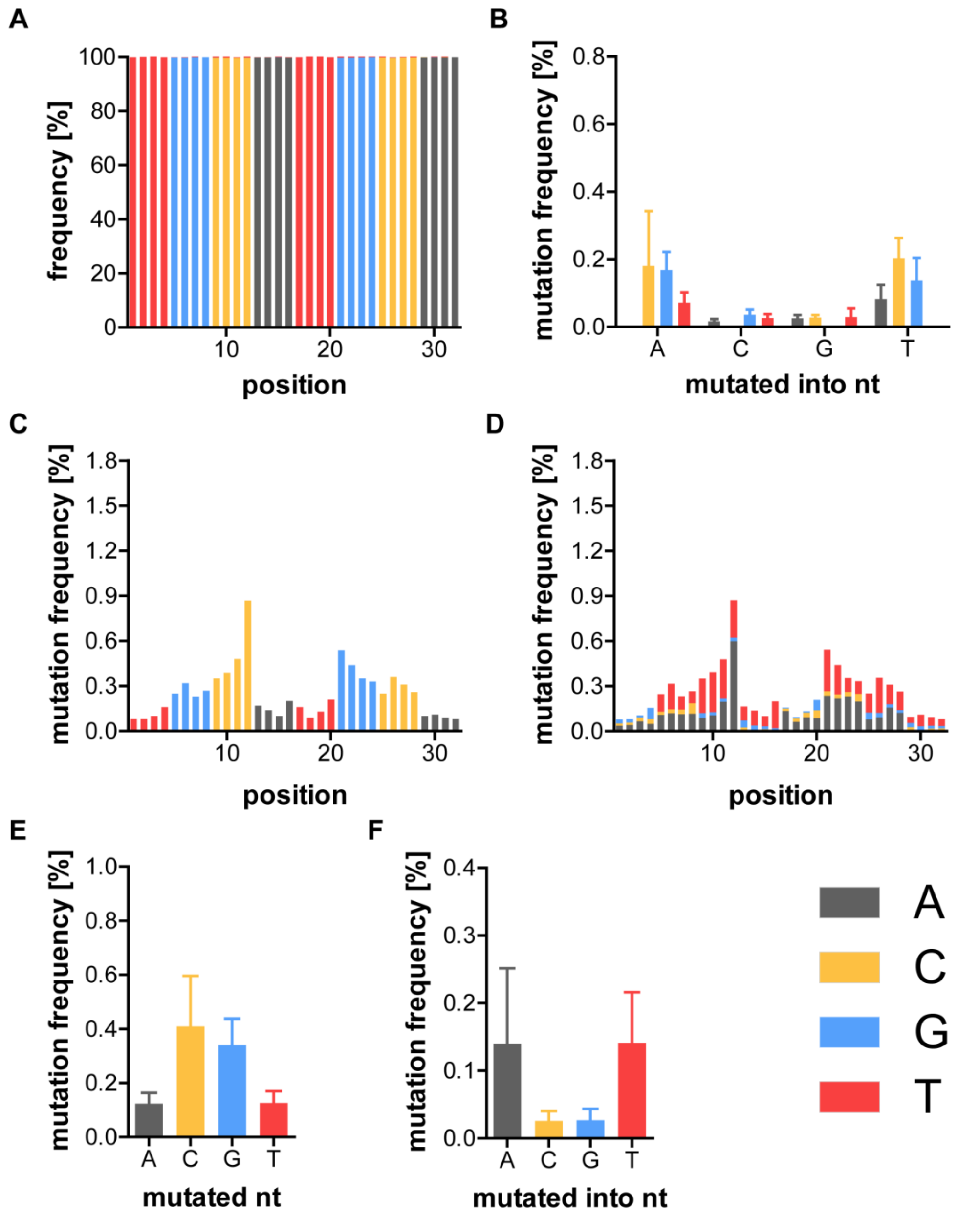

Figure S27: Mutation analysis of FT2-T4G4C4A4 after omission of shortened sequences

(**A**) Frequency of the different nucleotides at all positions of the random region. (**B**) Frequencies with which one nucleotide (denoted by colour) mutates into another nucleotide (denoted on the x-axis). Depicted is the mean and SD. Mutation frequency at all positions of the random region of (**C**) the original nucleotide and (**D**) the nucleotide that has been mutated into. Mutation frequencies of the original nucleotides (**E**) and the nucleotides that has been mutated into (**F**). Depicted is the mean and SD.

Figure S28

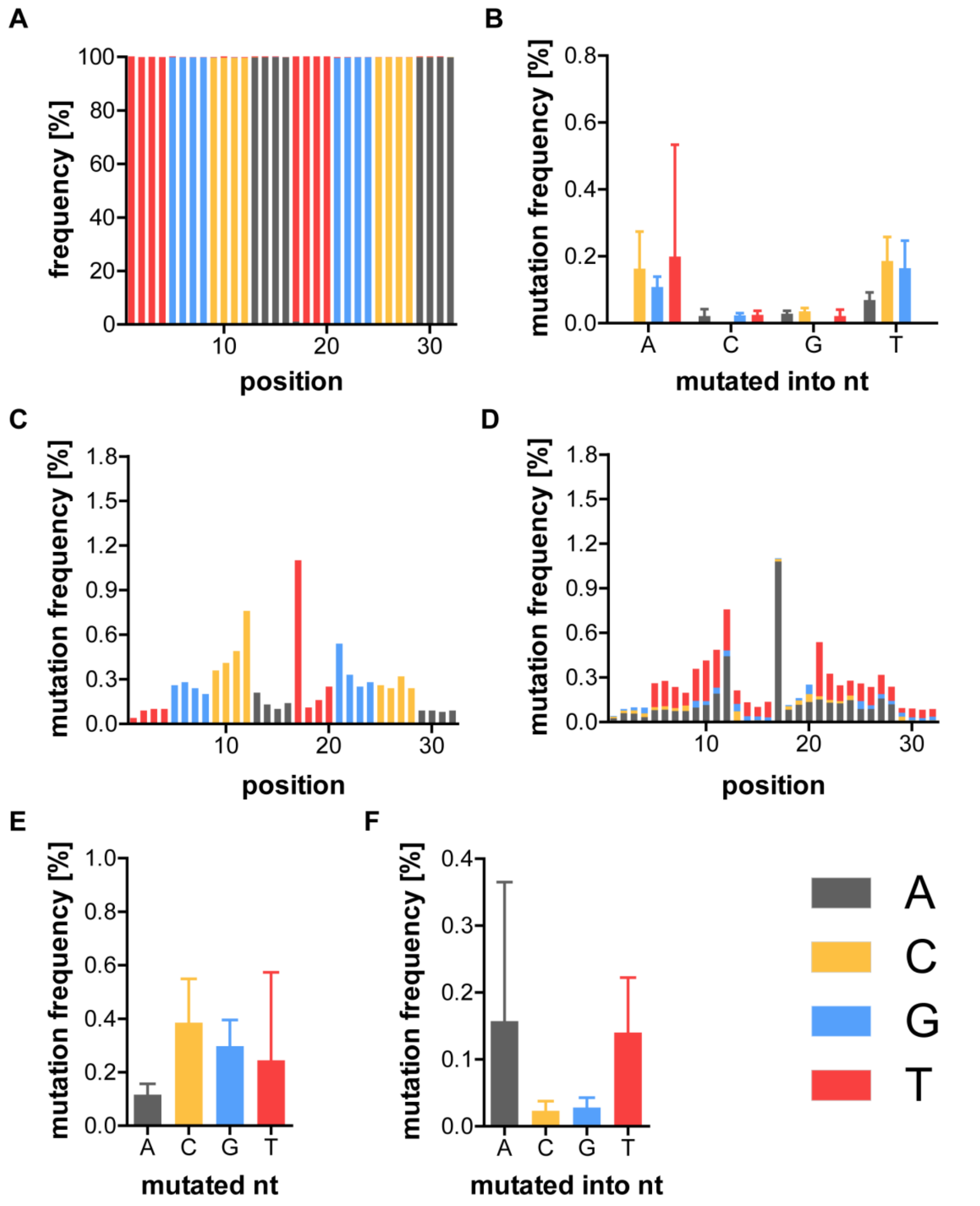

Figure S28: Mutation analysis of D3-T4G4C4A4 after omission of shortened sequences

(**A**) Frequency of the different nucleotides at all positions of the random region. (**B**) Frequencies with which one nucleotide (denoted by colour) mutates into another nucleotide (denoted on the x-axis). Depicted is the mean and SD. Mutation frequency at all positions of the random region of (**C**) the original nucleotide and (**D**) the nucleotide that has been mutated into. Mutation frequencies of the original nucleotides (**E**) and the nucleotides that has been mutated into (**F**). Depicted is the mean and SD.
